# Syntheses and crystal structures of four 4-(4-meth­oxy­phen­yl)piperazin-1-ium salts: tri­fluoro­acetate, 2,3,4,5,6-penta­fluoro­benzoate, 4-iodo­benzoate, and a polymorph with 4-methyl­benzoate

**DOI:** 10.1107/S2056989023002529

**Published:** 2023-03-23

**Authors:** Vinaya, Yeriyur B. Basavaraju, Hemmige S. Yathirajan, Sean Parkin

**Affiliations:** a Department of Studies in Chemistry, University of Mysore, Manasagangotri, Mysuru-570 006, India; b Department of Chemistry, University of Kentucky, Lexington, KY, 40506-0055, USA; Katholieke Universiteit Leuven, Belgium

**Keywords:** crystal structure, piperazine, piperazinium salt, hydrogen bonding, iodine–iodine close contacts, polymorph

## Abstract

Syntheses and X-ray crystal structures of four 4-(4-meth­oxy­phen­yl)piperazin-1-ium (MeOPP) salts, with 2,2,2-tri­fluoro­acetate, C_11_H_17_N_2_O^+^·C_2_F_3_O_2_^−^ (**I**), 2,3,4,5,6-penta­fluoro­benzoate, C_11_H_17_N_2_O^+^·C_7_F_5_O_2_^−^·H_2_O (**II**), 4-iodo­benzoate C_11_H_17_N_2_O^+^·C_7_H_4_IO_2_^−^·H_2_O (**III**), and 4-methyl­benzoate, C_11_H_17_N_2_O^+^·C_8_H_7_O_2_^−^·H_2_O (**IV**) anions are presented. The salts form directly from equimolar qu­anti­ties of *N*-(4-meth­oxy­phen­yl)piperazine and the corresponding organic acid in methanol and crystallize from 1:1 methanol/ethyl acetate. Salt **I** is anhydrous whereas **II**, **III**, and **IV** are all monohydrates. In all cases, the MeOPP cation conformation is determined by the torsion about the N—C bond between the piperazinium and 4-meth­oxy­benzene rings. Crystal packing in each structure is largely dictated by N—H⋯O and (in **II**, **III**, and **IV**) O—H⋯O hydrogen bonds, although each also features weak C—H⋯O-type hydrogen bonds. Salt **II** also has π–π-stacking inter­actions between cation and anion arene rings, and **III** exhibits I⋯I close contacts.

## Abstract 2

The syntheses and low-temperature (90 K) crystal structures of four organic salts of 4-(4-meth­oxy­phen­yl)piperazin-1-ium are presented.

## 1. Chemical context

In recent years, *N*-(4-meth­oxy­phen­yl)piperazine (MeOPP) has emerged as an addition to the range of designer recreational drugs. As such, considerable effort has been invested in the development of methods for the detection both of MeOPP itself and of its metabolites; *N*-(4-hy­droxy­phen­yl)piperazine and 4-hy­droxy­aniline (Arbo *et al.*, 2012 ▸) in human fluids (Staack & Maurer, 2003 ▸; Staack *et al.*, 2004 ▸). MeOPP imparts euphoric stimulant properties, its actions on human physiology being similar to those of amphetamines (Staack & Maurer, 2005 ▸; Wohlfarth *et al.*, 2010 ▸), but it has a significantly lower potential for abuse (Nagai *et al.*, 2007 ▸). However, no therapeutic applications of MeOPP have been reported to date. In view of the reported psychoactive properties of MeOPP, coupled with the broad range of biological activities exhibited by piperazine derivatives in general (Asif, 2015 ▸; Brito *et al.*, 2019 ▸), we recently initiated a programme of study centred on *N*-(4-meth­oxy­phen­yl)piperazine derivatives. Thus far, we have reported the synthesis and structures of a series of 1-aroyl-4-(4-meth­oxy­phen­yl) piperazines (Kiran Kumar *et al.*, 2019*a* ▸). We have also reported a series of 4-meth­oxy­phenyl piperazin-1-ium salts formed with simple organic acids (Kiran Kumar *et al.*, 2019*b* ▸), and also reported crystal structures of the free-base compound *N*-(4-meth­oxy­phen­yl)piperazine (MeOPP) and three of its salts (Kiran Kumar *et al.*, 2020*a* ▸). More recently, we reported the crystal structures of 4-(4-meth­oxy­phen­yl)piperazin-1-ium 4-methyl­benzoate monohydrate and bis-[4-(4-meth­oxy­phen­yl)piperazin-1-ium] benzene-1,2-di­carb­oxyl­ate (Shankara Prasad *et al.*, 2022 ▸). 
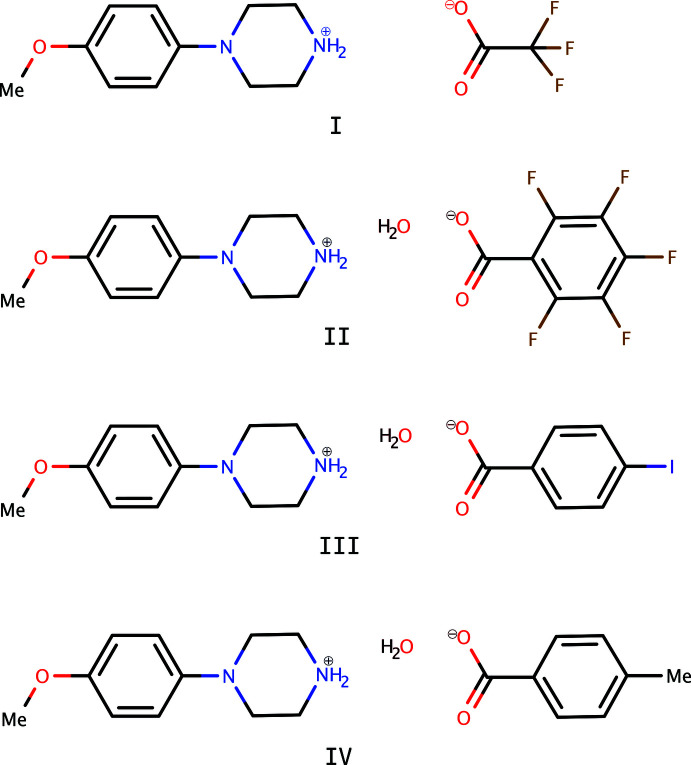


In view of the pharmacological importance of piperazines and the use of *N*-(4-meth­oxy­phen­yl)piperazine in particular, this paper presents the syntheses and crystal structures of *N*-(4-meth­oxy­phen­yl)piperazin-1-ium tri­fluoro­acetate, C_11_H_17_N_2_O^+^·C_2_F_3_O_2_^−^ (**I**), *N*-(4-meth­oxy­phen­yl)piperazin-1-ium 2,3,4,5,6-penta­fluoro­benzoate monohydrate, C_11_H_17_N_2_O^+^·C_7_F_5_O_2_^−^·H_2_O (**II**), *N*-(4-meth­oxy­phen­yl)piper­azin-1-ium 4-iodo­benzoate monohydrate, C_11_H_17_N_2_O^+^·C_7_H_4_IO_2_^−^·H_2_O (**III**) and a polymorph of *N*-(4-meth­oxy­phen­yl)piperazin-1-ium 4-methyl­benzoate monohydrate, C_11_H_17_N_2_O^+^·C_8_H_7_O_2_^−^·H_2_O (**IV**).

## 2. Structural commentary

**Figure 1:**
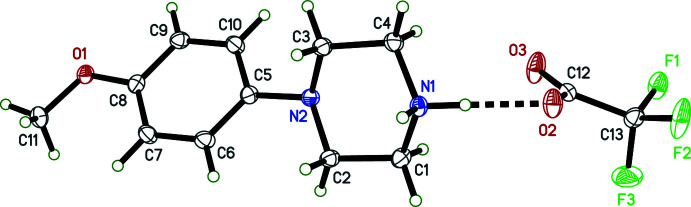
An ellipsoid plot (50% probability) of **I**. The dashed line denotes an N—H⋯O hydrogen bond. Hydrogen atoms are drawn as arbitrary circles.

Three of the four salts (see Figs. 1 ▸–4 ▸ ▸ ▸) crystallized as monohydrates; only **I** is anhydrous. Structure **III**, which is a much higher quality, low-temperature re-investigation of CSD entry KUJPUD [Kiran Kumar *et al.*, 2020*b* ▸; CSD = Cambridge Structural Database (Groom *et al.*, 2016 ▸)] contains three copies of the cation, anion, and water within its asymmetric unit (*i.e. Z*′ = 3), all others have *Z*′ = 1. Structure **IV** is a polymorph of CSD entry XEMCIF (Shankara Prasad *et al.*, 2022 ▸). The asymmetric units were chosen so as to make the N—H⋯O hydrogen-bond geometry between the cation and anion as similar as possible, *i.e.* with the equatorial H atom of the NH_2_^+^ group as donor (see section 3: *Supra­molecular features*). The overall conformations of the cations in **I**–**IV** are determined, in large part, by the twist of the N2—C5 bonds that connect the 4-meth­oxy­phenyl and piperazinium groups (Figs. 1 ▸–4 ▸ ▸ ▸). These twists, qu­anti­fied for example by the dihedral angle between the mean planes of the benzene ring (C5–C10) and the four carbon atoms (C1–C4) of the piperazinium rings, are 40.63 (5)° (**I**), 36.05 (4)° (**II**), 25.28 (13), 26.59 (12), and 24.82 (11)° (for **III**a,b,c, respectively), and 7.57 (8)° (**IV**), showing moderate variability across the four structures (Fig. 5 ▸). The geometry of the N2 atoms in each cation is non-planar; the sums of bond angles about N2 ranging from 337.46 (16)° in **I** to 342.4 (3)° for N2*C* in **III**c. In each structure, the 4-meth­oxy groups are close to coplanar with their attached benzene rings, the largest deviation out of plane being only 0.188 (4) Å for C11*C* in **III**c, which corresponds to a C9*C*—C8*C*—O1*C*—C11*C* torsion angle of 172.3 (2)°.

The conformation of the tri­fluoro­acetate anion in **I**, is largely unremarkable, having a dihedral angle between the plane of the carboxyl­ate group and the plane formed by atoms C12, C13, and F3 of 89.29 (12)°. In the substituted benzoate anions of **II**, **III**, and **IV**, the dihedral angles between the carboxyl­ate groups and the benzene rings are 43.28 (5)° (**II**), 3.8 (2)°, 7.46 (19)°, and 23.6 (2)° (**III**a,b,c, respectively) and 8.60 (11)° (**IV**).

## 3. Supra­molecular features

Strong hydrogen bonds are the dominant inter­molecular inter­actions in each of the four salts. All other hydrogen-bond-type inter­actions are weak. Salt **II** has weak π–π inter­actions and **III** has I⋯I contacts, as described below.

The hydrogen bonding in **I** is the simplest of the four salts. There are only two N—H⋯O hydrogen bonds, the shortest being N1—H1*NA*⋯O2, at *d_D⋯A_* = 2.7084 (13) Å. In addition, N1—H1*NB*⋯O3^i^ (symmetry code as per Table 1 ▸) at *d_D⋯A_* = 2.8329 (14) Å, connects cations and anions into chains that extend parallel to its crystallographic *b* axis, with inversion-related chains running anti-parallel (Fig. 6 ▸). Other than a few C—H⋯O and C—H⋯F close contacts (also listed in Table 1 ▸), there are no other significant inter-species inter­actions.

In **II**, the chosen asymmetric unit includes N1—H1*NA*⋯O2 as the shortest hydrogen bond, at 2.7464 (12) Å. The cation also hydrogen bonds to an inversion-related anion *via* N1—H1*NB*⋯O3^i^ (symmetry operator as per Table 2 ▸), *d_D⋯A_* = 2.7692 (12) Å. The water mol­ecule acts as donor in two strong O1*W*—H1*W*1⋯O3 (same asymmetric unit) and O1*W*—H2*W*1⋯O2^ii^ (2_1_ screw-related, as per Table 2 ▸) hydrogen bonds, and as acceptor in two weak C—H⋯O_water_ inter­actions, as listed in Table 2 ▸. There are a few much weaker C—H⋯F close contacts (Table 2 ▸). The hydrogen bonding in **II** is augmented by π–π stacking of the cation benzene ring with 2_1_ screw (−*x* + 1, *y* − 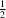 , −*z* + 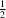 ) and inversion-related (1 − *x*, 1 − *y*, 1 − *z*) anion penta­fluoro­benzene rings. However, the fluorinated rings are 6.03 (3)° out of co-planarity with the MeOPP arene ring and the stacking is offset, leading to centroid–centroid distances of 3.567 (2) Å. The net result is a complicated double-layered structure that extends parallel to the *bc* plane, a slice through which is shown in Fig. 7 ▸.

**Figure 3:**
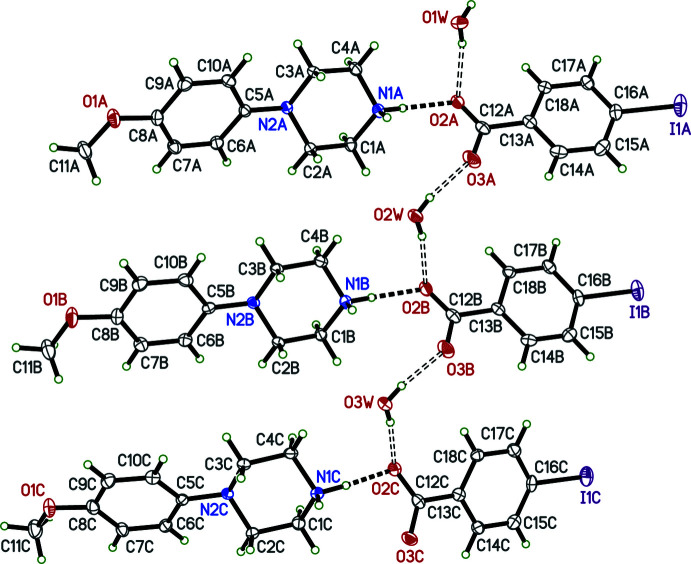
An ellipsoid plot (50% probability) of **III**. The solid dashed lines denote N—H⋯O hydrogen bonds, while the open dashed lines represent O—H⋯O hydrogen bonds. Hydrogen atoms are drawn as arbitrary circles.

**Figure 5:**
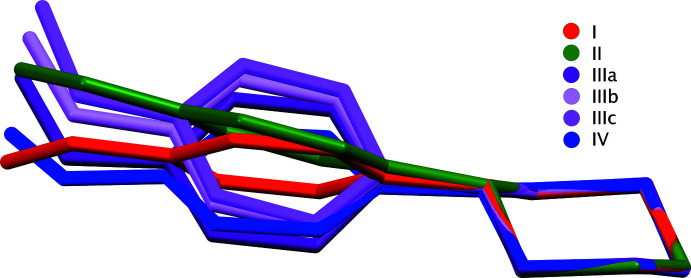
An overlay of six 4-MeOPP cations (least-squares fit of piperazinium ring atoms), showing the variability of MeOPP conformation across structures **I**–**IV**.

**Figure 6:**
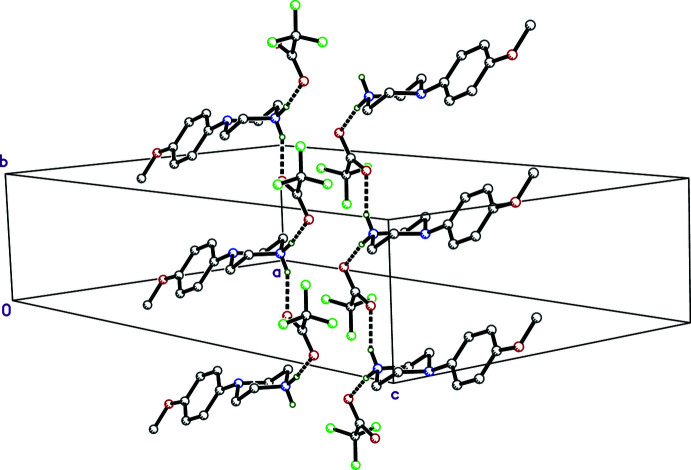
A partial packing plot of **I** showing hydrogen-bonded chains (solid dashed lines) parallel to the *b*-axis. Hydrogen atoms not involved in hydrogen bonds are omitted.

**Figure 7:**
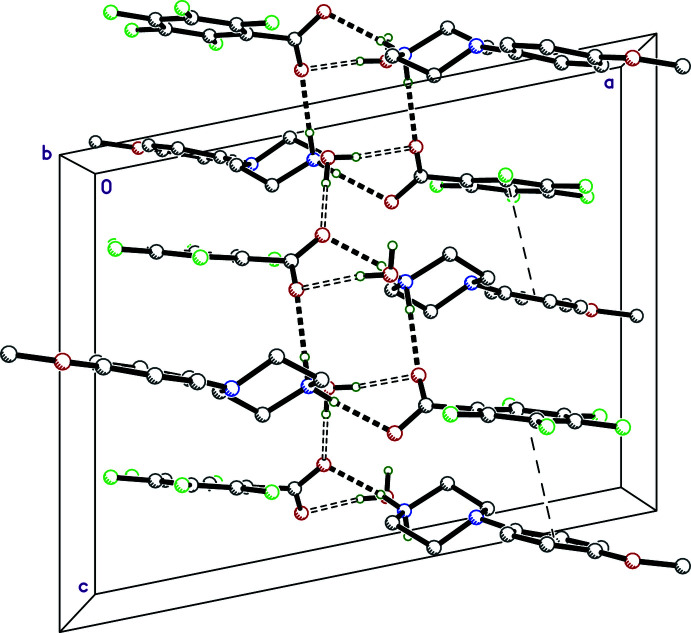
A partial packing plot of **II** viewed down the *b*-axis showing a slice through the N—H⋯O (solid dashed lines) and O—H⋯O (open dashed lines) hydrogen-bonded double layers. Thin dashed lines indicate π–π stacking of cation and anion arene rings. Hydrogen atoms not participating in hydrogen bonds are omitted.

**Figure 8:**
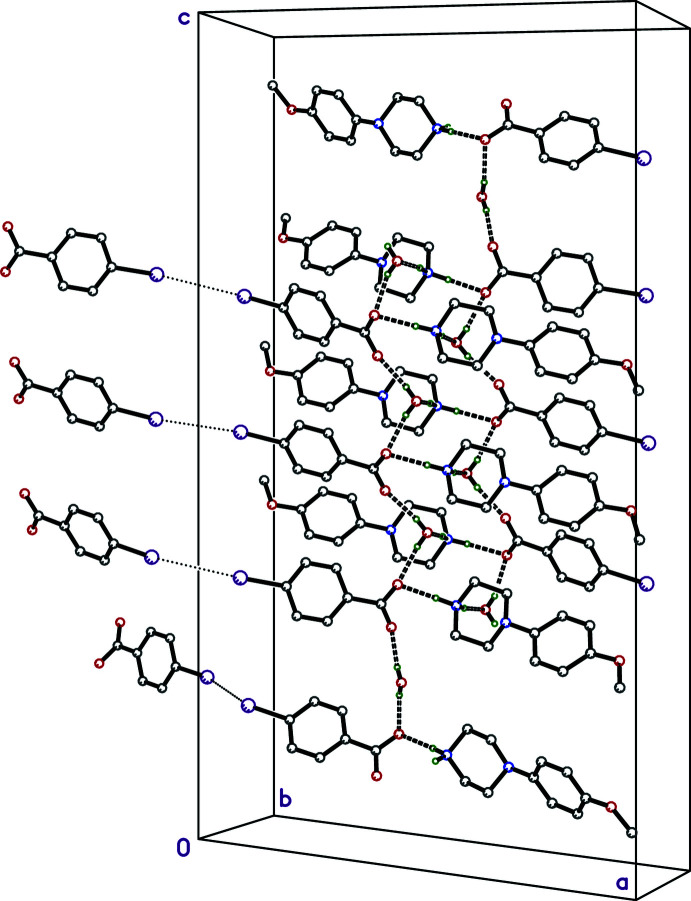
A partial packing plot of **III** viewed approximately down the *b*-axis showing a slice through the N—H⋯O (solid dashed lines) and O—H⋯O (open dashed lines) hydrogen-bonded double layers, which stack along the *b*-axis direction *via* I⋯I close contacts (dotted lines). Hydrogen atoms not involved in hydrogen bonds are omitted.

The asymmetric unit of **III** contains three crystallographically inequivalent groups of cation, anion, and water mol­ecules (Fig. 3 ▸). Within each group, the species are hydrogen bonded in a similar pattern to the asymmetric unit of **II**, and the cation–anion pairs are linked by the water mol­ecules. The asymmetric units are linked by O—H⋯O_water_ hydrogen bonds to glide-related (*x*, 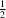 − *y*, 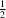 + *z* and *x*, − 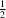 − *y*, 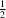 + *z*) equivalents parallel to *c* (Figs. 3 ▸ and 8 ▸). These connections lead to double layers parallel to the *ac* plane. Details of the hydrogen-bonding inter­actions are given in Table 3 ▸. The double-layers are themselves connected by I⋯I close contacts to an inversion-related asymmetric unit [*d*_I⋯I_ = 3.8586 (4) Å for I1*B*⋯I1*B* ^inv^ and 4.0444 (4) Å for I1*A*⋯I1*C* ^inv^ and I1*C*⋯I1*A* ^inv^ (inv = −*x*, −*y*, 1 − *z*)], also depicted in Fig. 8 ▸.

**Figure 9:**
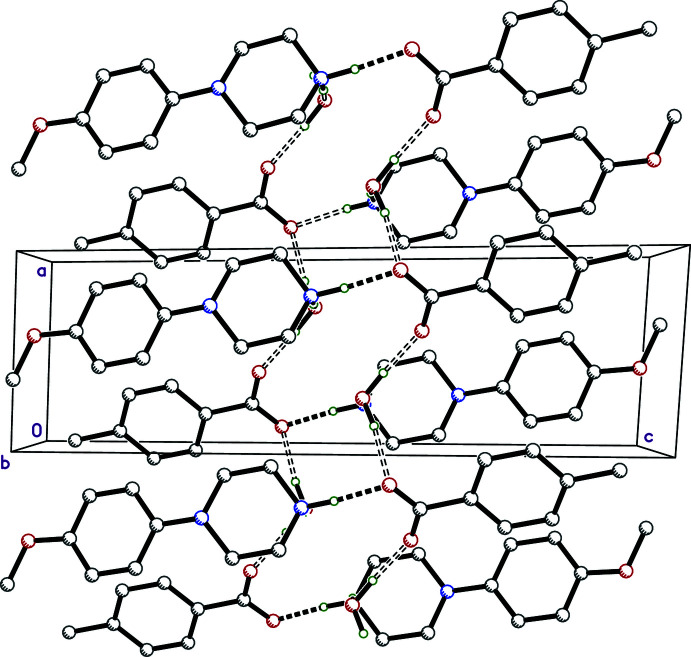
A packing plot of **IV** viewed down the *b*-axis, showing N—H⋯O (solid dashes) and O—H⋯O (open dashes) hydrogen bonds, which form bi-layered tapes in the *ac* plane that extend parallel to *a*. Hydrogen atoms not involved in hydrogen bonds are omitted.

In the structure of **IV**, a strong N1—H1*NA*⋯O2 [*d_D⋯A_* = 2.7391 (15) Å] hydrogen bond links the cation and anion. In combination with hydrogen bonds N1—H1*NB*⋯O1*W* ^i^, O1*W*—H1*W*⋯O3, and O1*W*—H2*W*⋯O2^iv^ (symmetry codes as per Table 4 ▸) involving water mol­ecules, bi-layered tapes in the *ac* plane extend along the *a*-axis direction (Fig. 9 ▸). There are no other noteworthy inter­actions other than a few C—H⋯O contacts, which are also listed in Table 4 ▸.

## 4. Database survey

A search of the Cambridge Structural Database (CSD, v5.43 plus updates to November 2022; Groom *et al.*, 2016 ▸) for a search fragment consisting of 1-phenyl­piperazine (without substituents) gave 1871 hits. With ‘any non-H atom’ substituted at the 4-position of the benzene ring, but all other carbons bearing hydrogen, a search found 225 matches, 46 of which had the *R*_2_H_2_^+^ piperazinium cation. This search fragment, but with a meth­oxy group added at the 4-position of the benzene ring, returned 20 structures. The parent mol­ecule, 4-MeOPP, is present as CSD entry IHILOD (Kiran Kumar *et al.*, 2020*a* ▸). CSD code EGUROO (Zia-ur-Rehman *et al.*, 2009 ▸) is the chloride salt of the 4-MeOPP cation and OMUXIG (Gharbi *et al.*, 2021 ▸) is a 4-MeOPP salt with Co(NCS)_4_^2–^ as its anion. The rest are all salts with a variety of organic anions: FOVPEO, FOVPOY, FOVPUE, FOVQAL, FOVQEP, FOVQIT, FOVQOZ, FOVQUF, FOVRAM, FOVREQ, FOVRIU, and FOVROA having been published by Kiran Kumar *et al.* (2019*a* ▸). Structures IHILUJ, IHIMAQ, & IHIMEU (along with IHILOD) were also published by Kiran Kumar *et al.* (2020*a* ▸), and XEMCIF and XEMCOL by Shankara Prasad *et al.* (2022 ▸). Entry KUJPUD, present as a *CSD Communication* (Kiran Kumar *et al.*, 2020*b* ▸), is a poor-quality room-temperature structure of the 4-iodo­benzoate, **III**. Similar related structures include the 1-aroyl-4-(4-meth­oxy­phen­yl) piperazines VONFOW, VONGAJ, VONGEN, VONGIR, VONGOX, & VONGUD (Kiran Kumar *et al.*, 2019*b* ▸).

## 5. Synthesis and crystallization

All reagents were obtained commercially and were used as received. For the synthesis of the salts, equimolar qu­anti­ties (0.52 mmol of each component) of *N*-(4-meth­oxy­phen­yl)piperazine (100 mg) (from Sigma-Aldrich) and either tri­fluoro­acetic acid (60 mg, **I**), penta­fluoro­benzoic acid (110 mg, **II**), 4-iodo­benzoic acid (129 mg, **III**), or 4-methyl­benzoic acid (71 mg, **IV**) were separately dissolved in methanol (10 ml). The two solutions were mixed and stirred briefly at 333 K and then set aside to crystallize, giving the solid products **I** to **IV** after a few days. The products were collected by filtration and then dried in air (**I**: yield 80%, m.p. 390–392 K; **II**: yield 75%, m.p. 375–377 K; **III**: yield 85%, m.p. 426–428 K; **IV**: yield 70%, m.p. 406–408 K). Crystals of compounds **I** to **IV** suitable for single-crystal X-ray diffraction were grown by slow evaporation, at ambient temperature and in the presence of air, of solutions in methanol:ethyl acetate (initial composition 1:1, *v*/*v*).

## 6. Refinement

Crystal data, data collection, and refinement statistics are given in Table 5 ▸. All hydrogen atoms were located in difference-Fourier maps. Those bound to nitro­gen or oxygen were refined freely, while carbon-bound hydrogens were included in the refinement using riding models with constrained distances set to 0.95 Å (C*sp* ^2^H), 0.99 Å (*R*_2_CH_2_), and 0.98 Å (*R*CH_3_) using *U*_iso_(H) values constrained to 1.2*U*_eq_ or 1.5*U*_eq_ (methyl only) of the attached carbon atom.

## Supplementary Material

Crystal structure: contains datablock(s) I, II, III, IV, global. DOI: 10.1107/S2056989023002529/vm2279sup1.cif

Structure factors: contains datablock(s) I. DOI: 10.1107/S2056989023002529/vm2279Isup2.hkl

Structure factors: contains datablock(s) II. DOI: 10.1107/S2056989023002529/vm2279IIsup3.hkl

Structure factors: contains datablock(s) III. DOI: 10.1107/S2056989023002529/vm2279IIIsup4.hkl

Structure factors: contains datablock(s) IV. DOI: 10.1107/S2056989023002529/vm2279IVsup5.hkl

CCDC references: 2248701, 2248700, 2248699, 2248698

Additional supporting information:  crystallographic information; 3D view; checkCIF report

## Figures and Tables

**Figure 2:**
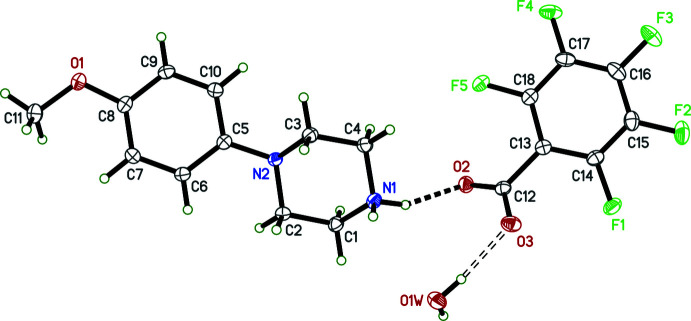
An ellipsoid plot (50% probability) of **II**. The solid dashed line denotes an N—H⋯O hydrogen bond, while the open dashed line shows an O—H⋯O hydrogen bond. Hydrogen atoms are drawn as arbitrary circles.

**Figure 4:**
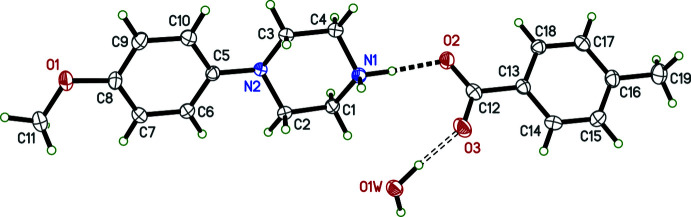
An ellipsoid plot (50% probability) of **IV**. The solid dashed line denotes an N—H⋯O hydrogen bond, while the open dashed line shows an O—H⋯O hydrogen bond. Hydrogen atoms are drawn as arbitrary circles.

**Table 1:** Hydrogen-bond geometry (Å, °) for **I** [Chem scheme1]

*D*—H⋯*A*	*D*—H	H⋯*A*	*D*⋯*A*	*D*—H⋯*A*
N1—H1*A*⋯O2	0.937 (16)	1.777 (16)	2.7084 (13)	172.1 (15)
N1—H1*B*⋯O3^i^	0.901 (16)	1.982 (16)	2.8329 (14)	156.7 (13)
C3—H3*B*⋯O1^ii^	0.99	2.55	3.2230 (14)	125
C4—H4*B*⋯F1^iii^	0.99	2.62	3.3162 (14)	127
C4—H4*B*⋯O2^iv^	0.99	2.51	3.1709 (14)	124
C11—H11*A*⋯F3^v^	0.98	2.59	3.2796 (15)	128

Symmetry codes: (i) 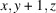 ; (ii) 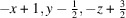 ; (iii) 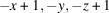 ; (iv) 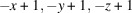 ; (v) 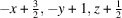 .

**Table 2:** Hydrogen-bond geometry (Å, °) for **II** [Chem scheme1]

*D*—H⋯*A*	*D*—H	H⋯*A*	*D*⋯*A*	*D*—H⋯*A*
N1—H1*A*⋯O2	0.912 (16)	1.886 (16)	2.7464 (12)	156.5 (14)
N1—H1*B*⋯O3^i^	0.882 (15)	1.909 (15)	2.7692 (12)	164.9 (13)
C1—H1*C*⋯F5^ii^	0.99	2.62	3.2261 (12)	119
C3—H3*B*⋯O1*W* ^iii^	0.99	2.65	3.5235 (14)	148
C4—H4*A*⋯O2^iv^	0.99	2.57	3.4812 (13)	153
C10—H10⋯O1*W* ^iii^	0.95	2.41	3.3581 (14)	177
C11—H11*A*⋯F3^v^	0.98	2.55	3.3961 (15)	144
C11—H11*B*⋯F2^vi^	0.98	2.51	3.2436 (15)	131
O1*W*—H1*W*⋯O3	0.854 (19)	1.978 (19)	2.8222 (12)	170.0 (17)
O1*W*—H2*W*⋯O2^ii^	0.899 (18)	1.936 (19)	2.8319 (12)	173.7 (16)

Symmetry codes: (i) 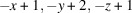 ; (ii) 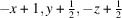 ; (iii) 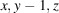 ; (iv) 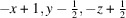 ; (v) 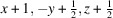 ; (vi) 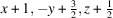 .

**Table 3:** Hydrogen-bond geometry (Å, °) for **III** [Chem scheme1]

*D*—H⋯*A*	*D*—H	H⋯*A*	*D*⋯*A*	*D*—H⋯*A*
O1*W*—H1*W*1⋯O3*C* ^i^	0.77 (2)	1.93 (2)	2.696 (3)	173 (3)
O1*W*—H2*W*1⋯O2*A*	0.77 (2)	1.96 (2)	2.700 (3)	162 (3)
O2*W*—H1*W*2⋯O3*A*	0.77 (1)	1.86 (2)	2.626 (2)	170 (3)
O2*W*—H2*W*2⋯O2*B*	0.77 (2)	1.97 (2)	2.733 (3)	168 (3)
O3*W*—H1*W*3⋯O3*B*	0.77 (2)	1.87 (2)	2.636 (2)	172 (3)
O3*W*—H2*W*3⋯O2*C*	0.78 (1)	1.94 (2)	2.706 (3)	172 (3)
N1*A*—H1*AB*⋯O3*W* ^ii^	0.94 (3)	1.82 (3)	2.754 (3)	172 (3)
N1*A*—H1*AA*⋯O2*A*	0.90 (3)	1.89 (3)	2.776 (3)	168 (3)
C1*A*—H1*AC*⋯O2*W*	0.99	2.57	3.362 (3)	136
C2*A*—H2*AB*⋯O3*B* ^ii^	0.99	2.51	3.498 (3)	175
C4*A*—H4*AA*⋯O2*C* ^iii^	0.99	2.47	3.441 (3)	166
C7*A*—H7*A*⋯I1*B* ^iii^	0.95	3.32	4.212 (2)	156
C11*A*—H11*A*⋯O1*B* ^iv^	0.98	2.51	3.225 (4)	130
N1*B*—H1*BA*⋯O2*B*	0.91 (3)	1.87 (3)	2.780 (3)	174 (3)
N1*B*—H1*BB*⋯O2*W* ^ii^	0.92 (3)	1.85 (3)	2.768 (3)	176 (2)
C1*B*—H1*BC*⋯O3*W*	0.99	2.44	3.229 (3)	136
C2*B*—H2*BB*⋯O3*A* ^ii^	0.99	2.63	3.619 (3)	174
C4*B*—H4*BA*⋯O2*B* ^iii^	0.99	2.51	3.491 (3)	170
C4*B*—H4*BB*⋯O2*W*	0.99	2.52	3.260 (3)	131
C9*B*—H9*B*⋯I1*B* ^ii^	0.95	3.25	4.020 (3)	139
N1*C*—H1*CA*⋯O2*C*	0.96 (3)	1.78 (3)	2.732 (3)	172 (3)
N1*C*—H1*CA*⋯O3*C*	0.96 (3)	2.64 (3)	3.297 (3)	126 (2)
N1*C*—H1*CB*⋯O1*W* ^ii^	0.83 (3)	1.96 (3)	2.781 (3)	172 (3)
C1*C*—H1*CC*⋯O1*W* ^v^	0.99	2.39	3.299 (3)	152
C4*C*—H4*CA*⋯O2*A* ^iii^	0.99	2.45	3.438 (3)	174
C9*C*—H9*C*⋯I1*A* ^ii^	0.95	3.29	4.013 (3)	135

Symmetry codes: (i) 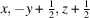 ; (ii) 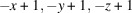 ; (iii) 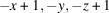 ; (iv) 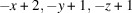 ; (v) 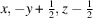 .

**Table 4:** Hydrogen-bond geometry (Å, °) for **IV** [Chem scheme1]

*D*—H⋯*A*	*D*—H	H⋯*A*	*D*⋯*A*	*D*—H⋯*A*
N1—H1*A*⋯O2	1.028 (17)	1.714 (17)	2.7391 (15)	174.1 (15)
N1—H1*B*⋯O1*W* ^i^	0.939 (16)	1.873 (17)	2.7977 (16)	167.8 (14)
C1—H1*C*⋯O1*W*	0.99	2.46	3.2617 (17)	138
C2—H2*B*⋯O3^i^	0.99	2.52	3.5088 (17)	174
C4—H4*A*⋯O2^ii^	0.99	2.55	3.5294 (17)	169
C4—H4*B*⋯O1*W* ^iii^	0.99	2.54	3.3184 (17)	135
O1*W*—H1*W*⋯O3	0.97 (2)	1.67 (2)	2.6418 (14)	176.5 (18)
O1*W*—H2*W*⋯O2^iv^	0.94 (2)	1.83 (2)	2.7626 (15)	170.5 (17)

Symmetry codes: (i) 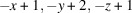 ; (ii) 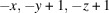 ; (iii) 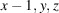 ; (iv) 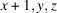 .

**Table 5:** Experimental details

	**I**	**II**	**III**	**IV**
Crystal data				
Chemical formula	C_11_H_17_N_2_O^+^·C_2_F_3_O_2_^−^	C_11_H_17_N_2_O^+^·C_7_F_5_O_2_^−^·H_2_O	C_11_H_17_N_2_O^+^·C_7_H_4_IO_2_^−^·H_2_O	C_11_H_17_N_2_O^+^·C_8_H_7_O_2_^−^·H_2_O
*M*_r_	306.28	422.35	458.28	346.42
Crystal system, space group	Orthorhombic, *P* *b* *c* *a*	Monoclinic, *P*2_1_/*c*	Monoclinic, *P*2_1_/*c*	Triclinic, *P* 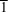
Temperature (K)	90	90	90	90
*a*, *b*, *c* (Å)	16.2967 (9), 6.0887 (2), 28.4139 (15)	16.9733 (7), 8.3512 (4), 13.2980 (4)	20.4117 (18), 7.4255 (6), 36.796 (3)	6.1481 (13), 7.3467 (12), 19.980 (4)
α, β, γ (°)	90, 90, 90	90, 101.494 (1), 90	90, 92.970 (3), 90	80.190 (6), 86.089 (5), 82.843 (6)
*V* (Å^3^)	2819.4 (2)	1847.16 (13)	5569.6 (8)	881.3 (3)
*Z*	8	4	12	2
Radiation type	Mo *K*α	Mo *K*α	Mo *K*α	Mo *K*α
μ (mm^−1^)	0.13	0.14	1.75	0.09
Crystal size (mm)	0.24 × 0.18 × 0.11	0.30 × 0.29 × 0.14	0.30 × 0.13 × 0.03	0.29 × 0.21 × 0.02

Data collection				
Diffractometer	Bruker D8 Venture dual source	Bruker D8 Venture dual source	Bruker D8 Venture dual source	Bruker D8 Venture dual source
Absorption correction	Multi-scan (*SADABS*; Krause *et al.*, 2015 ▸)	Multi-scan (*SADABS*; Krause *et al.*, 2015 ▸)	Multi-scan (*SADABS*; Krause *et al.*, 2015 ▸)	Multi-scan (*SADABS*; Krause *et al.*, 2015 ▸)
*T*_min_, *T*_max_	0.847, 0.958	0.919, 0.971	0.726, 0.862	0.877, 0.959
No. of measured, independent and observed [*I* > 2σ(*I*)] reflections	26032, 3243, 2802	30724, 4238, 3793	72181, 12876, 10003	23917, 4059, 3137
*R*_int_	0.038	0.031	0.056	0.041
(sin θ/λ)_max_ (Å^−1^)	0.651	0.650	0.653	0.652

Refinement				
*R*[*F* ^2^ > 2σ(*F* ^2^)], *wR*(*F* ^2^), *S*	0.034, 0.087, 1.05	0.031, 0.083, 1.03	0.033, 0.069, 1.03	0.038, 0.080, 1.07
No. of reflections	3243	4238	12876	4059
No. of parameters	200	280	729	245
No. of restraints	0	0	12	0
H-atom treatment	H atoms treated by a mixture of independent and constrained refinement	H atoms treated by a mixture of independent and constrained refinement	H atoms treated by a mixture of independent and constrained refinement	H atoms treated by a mixture of independent and constrained refinement
Δρ_max_, Δρ_min_ (e Å^−3^)	0.39, −0.22	0.34, −0.19	0.84, −0.81	0.19, −0.18

Computer programs: *APEX3* (Bruker, 2016 ▸), *SHELXT* (Sheldrick, 2015*a* ▸), *SHELXL2019/2* (Sheldrick, 2015*b* ▸), *XP* in *SHELXTL* (Sheldrick, 2008 ▸), *Mercury* (Macrae *et al.*, 2020 ▸), *SHELX* (Sheldrick, 2008 ▸) and *publCIF* (Westrip, 2010 ▸).

## supplementary crystallographic information

### 4-(4-Methoxyphenyl)piperazin-1-ium 2,2,2-trifluoroacetate (I) Crystal data

**Table:**

C_11_H_17_N_2_O^+^·C_2_F_3_O_2_^−^	*D*_x_ = 1.443 Mg m^−^^3^
*M**_r_* = 306.28	Mo *K*α radiation, λ = 0.71073 Å
Orthorhombic, *P**b**c**a*	Cell parameters from 9961 reflections
*a* = 16.2967 (9) Å	θ = 2.5–27.5°
*b* = 6.0887 (2) Å	µ = 0.13 mm^−^^1^
*c* = 28.4139 (15) Å	*T* = 90 K
*V* = 2819.4 (2) Å^3^	Solvent-rounded block, colourless
*Z* = 8	0.24 × 0.18 × 0.11 mm
*F*(000) = 1280	

### 4-(4-Methoxyphenyl)piperazin-1-ium 2,2,2-trifluoroacetate (I) Data collection

**Table:**

Bruker D8 Venture dual source diffractometer	3243 independent reflections
Radiation source: microsource	2802 reflections with *I* > 2σ(*I*)
Detector resolution: 7.41 pixels mm^-1^	*R*_int_ = 0.038
φ and ω scans	θ_max_ = 27.6°, θ_min_ = 1.9°
Absorption correction: multi-scan (*SADABS*; Krause *et al*., 2015)	*h* = −20→21
*T*_min_ = 0.847, *T*_max_ = 0.958	*k* = −7→7
26032 measured reflections	*l* = −36→37

### 4-(4-Methoxyphenyl)piperazin-1-ium 2,2,2-trifluoroacetate (I) Refinement

**Table:**

Refinement on *F*^2^	Secondary atom site location: difference Fourier map
Least-squares matrix: full	Hydrogen site location: mixed
*R*[*F*^2^ > 2σ(*F*^2^)] = 0.034	H atoms treated by a mixture of independent and constrained refinement
*wR*(*F*^2^) = 0.087	*w* = 1/[σ^2^(*F*_o_^2^) + (0.0351*P*)^2^ + 1.3982*P*] where *P* = (*F*_o_^2^ + 2*F*_c_^2^)/3
*S* = 1.05	(Δ/σ)_max_ = 0.001
3243 reflections	Δρ_max_ = 0.39 e Å^−^^3^
200 parameters	Δρ_min_ = −0.22 e Å^−^^3^
0 restraints	Extinction correction: SHELXL-2019/2 (Sheldrick 2015b), Fc^*^=kFc[1+0.001xFc^2^λ^3^/sin(2θ)]^-1/4^
Primary atom site location: structure-invariant direct methods	Extinction coefficient: 0.0023 (7)

### 4-(4-Methoxyphenyl)piperazin-1-ium 2,2,2-trifluoroacetate (I) Special details

**Table:**

Experimental. The crystal was mounted using polyisobutene oil on the tip of a fine glass fibre, which was fastened in a copper mounting pin with electrical solder. It was placed directly into the cold gas stream of a liquid-nitrogen based cryostat (Hope, 1994; Parkin & Hope, 1998). Diffraction data were collected with the crystal at 90K, which is standard practice in this laboratory for the majority of flash-cooled crystals.
Geometry. All esds (except the esd in the dihedral angle between two l.s. planes) are estimated using the full covariance matrix. The cell esds are taken into account individually in the estimation of esds in distances, angles and torsion angles; correlations between esds in cell parameters are only used when they are defined by crystal symmetry. An approximate (isotropic) treatment of cell esds is used for estimating esds involving l.s. planes.
Refinement. Refinement progress was checked using *Platon* (Spek, 2020) and by an *R*-tensor (Parkin, 2000). The final model was further checked with the IUCr utility *checkCIF*.

### 4-(4-Methoxyphenyl)piperazin-1-ium 2,2,2-trifluoroacetate (I) Fractional atomic coordinates and isotropic or equivalent isotropic displacement parameters (Å^2^)

**Table:**

	*x*	*y*	*z*	*U*_iso_*/*U*_eq_	
O1	0.64348 (5)	0.88138 (14)	0.84741 (3)	0.01761 (19)	
N1	0.57880 (6)	0.57351 (17)	0.56220 (3)	0.0147 (2)	
H1A	0.5746 (10)	0.487 (3)	0.5350 (6)	0.029 (4)*	
H1B	0.5680 (9)	0.713 (3)	0.5535 (5)	0.023 (4)*	
N2	0.61770 (6)	0.60018 (16)	0.66037 (3)	0.0155 (2)	
C1	0.66484 (7)	0.5639 (2)	0.57944 (4)	0.0179 (2)	
H1C	0.702091	0.626714	0.555436	0.022*	
H1D	0.680835	0.408985	0.584747	0.022*	
C2	0.67297 (7)	0.6921 (2)	0.62512 (4)	0.0172 (2)	
H2A	0.730261	0.684243	0.636575	0.021*	
H2B	0.659190	0.848350	0.619580	0.021*	
C3	0.53255 (7)	0.6192 (2)	0.64390 (4)	0.0179 (2)	
H3A	0.519051	0.775947	0.638751	0.022*	
H3B	0.494967	0.561212	0.668281	0.022*	
C4	0.52025 (7)	0.4935 (2)	0.59861 (4)	0.0181 (2)	
H4A	0.529255	0.334880	0.604256	0.022*	
H4B	0.463242	0.513595	0.587369	0.022*	
C5	0.62749 (7)	0.68174 (19)	0.70730 (4)	0.0155 (2)	
C6	0.67365 (7)	0.8657 (2)	0.71851 (4)	0.0186 (3)	
H6	0.700715	0.944071	0.694126	0.022*	
C7	0.68114 (7)	0.9382 (2)	0.76507 (4)	0.0186 (2)	
H7	0.713564	1.063514	0.772171	0.022*	
C8	0.64107 (7)	0.82630 (19)	0.80068 (4)	0.0154 (2)	
C9	0.59454 (7)	0.6405 (2)	0.78982 (4)	0.0176 (2)	
H9	0.567039	0.563175	0.814201	0.021*	
C10	0.58821 (7)	0.5683 (2)	0.74383 (4)	0.0182 (2)	
H10	0.556949	0.440604	0.736930	0.022*	
C11	0.68848 (8)	1.0742 (2)	0.85963 (4)	0.0216 (3)	
H11A	0.683518	1.100895	0.893519	0.032*	
H11B	0.666423	1.200190	0.842301	0.032*	
H11C	0.746404	1.053795	0.851455	0.032*	
F1	0.62003 (5)	−0.25022 (11)	0.46795 (3)	0.02609 (19)	
F2	0.60056 (6)	0.00973 (14)	0.41859 (3)	0.0388 (2)	
F3	0.71197 (5)	−0.00256 (16)	0.45876 (4)	0.0485 (3)	
O2	0.57475 (6)	0.29613 (14)	0.48772 (3)	0.0226 (2)	
O3	0.59231 (7)	0.02571 (15)	0.54005 (3)	0.0290 (2)	
C12	0.59453 (7)	0.10857 (19)	0.50037 (4)	0.0151 (2)	
C13	0.63112 (8)	−0.0358 (2)	0.46090 (4)	0.0198 (3)	

### 4-(4-Methoxyphenyl)piperazin-1-ium 2,2,2-trifluoroacetate (I) Atomic displacement parameters (Å^2^)

**Table:**

	*U*^11^	*U*^22^	*U*^33^	*U*^12^	*U*^13^	*U*^23^
O1	0.0188 (4)	0.0216 (4)	0.0124 (4)	−0.0011 (3)	−0.0001 (3)	−0.0024 (3)
N1	0.0180 (5)	0.0138 (5)	0.0124 (4)	0.0007 (4)	−0.0012 (4)	0.0000 (4)
N2	0.0148 (5)	0.0202 (5)	0.0114 (4)	−0.0027 (4)	−0.0008 (3)	0.0002 (4)
C1	0.0156 (5)	0.0232 (6)	0.0150 (5)	0.0022 (5)	−0.0001 (4)	0.0000 (4)
C2	0.0146 (5)	0.0231 (6)	0.0138 (5)	−0.0026 (4)	0.0002 (4)	0.0011 (4)
C3	0.0145 (5)	0.0252 (6)	0.0141 (5)	−0.0023 (4)	−0.0008 (4)	−0.0013 (4)
C4	0.0178 (5)	0.0228 (6)	0.0137 (5)	−0.0044 (5)	−0.0009 (4)	−0.0002 (4)
C5	0.0151 (5)	0.0184 (5)	0.0128 (5)	0.0001 (4)	−0.0015 (4)	0.0003 (4)
C6	0.0195 (6)	0.0218 (6)	0.0147 (5)	−0.0048 (5)	0.0004 (4)	0.0024 (4)
C7	0.0199 (6)	0.0188 (6)	0.0169 (5)	−0.0050 (5)	−0.0019 (4)	−0.0008 (4)
C8	0.0145 (5)	0.0186 (6)	0.0130 (5)	0.0028 (4)	−0.0016 (4)	−0.0009 (4)
C9	0.0169 (5)	0.0209 (6)	0.0150 (5)	−0.0020 (5)	0.0007 (4)	0.0027 (4)
C10	0.0191 (6)	0.0185 (6)	0.0170 (5)	−0.0044 (5)	−0.0012 (4)	0.0002 (4)
C11	0.0234 (6)	0.0235 (6)	0.0181 (5)	−0.0025 (5)	−0.0022 (5)	−0.0047 (5)
F1	0.0437 (5)	0.0128 (4)	0.0218 (4)	0.0006 (3)	0.0057 (3)	−0.0020 (3)
F2	0.0776 (7)	0.0254 (4)	0.0134 (4)	0.0096 (4)	0.0024 (4)	−0.0011 (3)
F3	0.0268 (5)	0.0461 (6)	0.0725 (7)	−0.0087 (4)	0.0245 (4)	−0.0251 (5)
O2	0.0341 (5)	0.0161 (4)	0.0174 (4)	0.0053 (4)	−0.0032 (4)	−0.0013 (3)
O3	0.0552 (7)	0.0175 (4)	0.0144 (4)	0.0027 (4)	0.0036 (4)	0.0009 (3)
C12	0.0155 (5)	0.0144 (5)	0.0154 (5)	−0.0020 (4)	−0.0001 (4)	−0.0018 (4)
C13	0.0247 (6)	0.0155 (6)	0.0192 (6)	−0.0020 (5)	0.0054 (5)	0.0002 (4)

### 4-(4-Methoxyphenyl)piperazin-1-ium 2,2,2-trifluoroacetate (I) Geometric parameters (Å, º)

**Table:**

O1—C8	1.3701 (13)	C5—C6	1.3862 (16)
O1—C11	1.4269 (15)	C5—C10	1.4015 (16)
N1—C1	1.4864 (15)	C6—C7	1.3998 (16)
N1—C4	1.4893 (14)	C6—H6	0.9500
N1—H1A	0.937 (16)	C7—C8	1.3835 (16)
N1—H1B	0.901 (16)	C7—H7	0.9500
N2—C5	1.4318 (14)	C8—C9	1.3964 (17)
N2—C2	1.4587 (14)	C9—C10	1.3824 (16)
N2—C3	1.4692 (14)	C9—H9	0.9500
C1—C2	1.5205 (16)	C10—H10	0.9500
C1—H1C	0.9900	C11—H11A	0.9800
C1—H1D	0.9900	C11—H11B	0.9800
C2—H2A	0.9900	C11—H11C	0.9800
C2—H2B	0.9900	F1—C13	1.3333 (14)
C3—C4	1.5106 (15)	F2—C13	1.3306 (14)
C3—H3A	0.9900	F3—C13	1.3343 (15)
C3—H3B	0.9900	O2—C12	1.2399 (14)
C4—H4A	0.9900	O3—C12	1.2356 (14)
C4—H4B	0.9900	C12—C13	1.5445 (16)
			
C8—O1—C11	116.87 (9)	C6—C5—C10	118.42 (10)
C1—N1—C4	111.25 (9)	C6—C5—N2	123.69 (10)
C1—N1—H1A	108.6 (10)	C10—C5—N2	117.9 (1)
C4—N1—H1A	110 (1)	C5—C6—C7	121.28 (11)
C1—N1—H1B	108.1 (9)	C5—C6—H6	119.4
C4—N1—H1B	111.9 (9)	C7—C6—H6	119.4
H1A—N1—H1B	106.9 (13)	C8—C7—C6	119.65 (11)
C5—N2—C2	115.95 (9)	C8—C7—H7	120.2
C5—N2—C3	112.00 (9)	C6—C7—H7	120.2
C2—N2—C3	109.51 (9)	O1—C8—C7	125.08 (11)
N1—C1—C2	110.07 (9)	O1—C8—C9	115.34 (10)
N1—C1—H1C	109.6	C7—C8—C9	119.57 (10)
C2—C1—H1C	109.6	C10—C9—C8	120.46 (11)
N1—C1—H1D	109.6	C10—C9—H9	119.8
C2—C1—H1D	109.6	C8—C9—H9	119.8
H1C—C1—H1D	108.2	C9—C10—C5	120.61 (11)
N2—C2—C1	109.59 (9)	C9—C10—H10	119.7
N2—C2—H2A	109.8	C5—C10—H10	119.7
C1—C2—H2A	109.8	O1—C11—H11A	109.5
N2—C2—H2B	109.8	O1—C11—H11B	109.5
C1—C2—H2B	109.8	H11A—C11—H11B	109.5
H2A—C2—H2B	108.2	O1—C11—H11C	109.5
N2—C3—C4	110.90 (9)	H11A—C11—H11C	109.5
N2—C3—H3A	109.5	H11B—C11—H11C	109.5
C4—C3—H3A	109.5	O3—C12—O2	129.27 (11)
N2—C3—H3B	109.5	O3—C12—C13	116.19 (10)
C4—C3—H3B	109.5	O2—C12—C13	114.45 (10)
H3A—C3—H3B	108.0	F2—C13—F1	106.79 (10)
N1—C4—C3	109.93 (9)	F2—C13—F3	107.26 (11)
N1—C4—H4A	109.7	F1—C13—F3	106.81 (11)
C3—C4—H4A	109.7	F2—C13—C12	113.14 (10)
N1—C4—H4B	109.7	F1—C13—C12	113.32 (9)
C3—C4—H4B	109.7	F3—C13—C12	109.16 (10)
H4A—C4—H4B	108.2		
			
C4—N1—C1—C2	−55.67 (12)	C11—O1—C8—C7	2.48 (16)
C5—N2—C2—C1	170.74 (9)	C11—O1—C8—C9	−178.21 (10)
C3—N2—C2—C1	−61.35 (12)	C6—C7—C8—O1	−179.82 (11)
N1—C1—C2—N2	58.98 (12)	C6—C7—C8—C9	0.89 (18)
C5—N2—C3—C4	−168.97 (9)	O1—C8—C9—C10	−179.47 (10)
C2—N2—C3—C4	60.94 (12)	C7—C8—C9—C10	−0.12 (18)
C1—N1—C4—C3	54.44 (12)	C8—C9—C10—C5	−0.80 (18)
N2—C3—C4—N1	−56.90 (12)	C6—C5—C10—C9	0.91 (18)
C2—N2—C5—C6	12.65 (16)	N2—C5—C10—C9	−179.22 (11)
C3—N2—C5—C6	−114.02 (12)	O3—C12—C13—F2	151.49 (11)
C2—N2—C5—C10	−167.21 (10)	O2—C12—C13—F2	−31.49 (15)
C3—N2—C5—C10	66.12 (13)	O3—C12—C13—F1	29.73 (16)
C10—C5—C6—C7	−0.13 (18)	O2—C12—C13—F1	−153.24 (11)
N2—C5—C6—C7	−179.99 (11)	O3—C12—C13—F3	−89.16 (13)
C5—C6—C7—C8	−0.78 (18)	O2—C12—C13—F3	87.86 (13)

### 4-(4-Methoxyphenyl)piperazin-1-ium 2,2,2-trifluoroacetate (I) Hydrogen-bond geometry (Å, º)

**Table:**

*D*—H···*A*	*D*—H	H···*A*	*D*···*A*	*D*—H···*A*
N1—H1*A*···O2	0.937 (16)	1.777 (16)	2.7084 (13)	172.1 (15)
N1—H1*B*···O3^i^	0.901 (16)	1.982 (16)	2.8329 (14)	156.7 (13)
C3—H3*B*···O1^ii^	0.99	2.55	3.2230 (14)	125
C4—H4*B*···F1^iii^	0.99	2.62	3.3162 (14)	127
C4—H4*B*···O2^iv^	0.99	2.51	3.1709 (14)	124
C11—H11*A*···F3^v^	0.98	2.59	3.2796 (15)	128

Symmetry codes: (i) *x*, *y*+1, *z*; (ii) −*x*+1, *y*−1/2, −*z*+3/2; (iii) −*x*+1, −*y*, −*z*+1; (iv) −*x*+1, −*y*+1, −*z*+1; (v) −*x*+3/2, −*y*+1, *z*+1/2.

### 4-(4-Methoxyphenyl)piperazin-1-ium 2,3,4,5,6-pentafluorobenzoate monohydrate (II) Crystal data

**Table:**

C_11_H_17_N_2_O^+^·C_7_F_5_O_2_^−^·H_2_O	*F*(000) = 872
*M**_r_* = 422.35	*D*_x_ = 1.519 Mg m^−^^3^
Monoclinic, *P*2_1_/*c*	Mo *K*α radiation, λ = 0.71073 Å
*a* = 16.9733 (7) Å	Cell parameters from 9821 reflections
*b* = 8.3512 (4) Å	θ = 2.9–27.5°
*c* = 13.2980 (4) Å	µ = 0.14 mm^−^^1^
β = 101.494 (1)°	*T* = 90 K
*V* = 1847.16 (13) Å^3^	Colourless block, colourless
*Z* = 4	0.30 × 0.29 × 0.14 mm

### 4-(4-Methoxyphenyl)piperazin-1-ium 2,3,4,5,6-pentafluorobenzoate monohydrate (II) Data collection

**Table:**

Bruker D8 Venture dual source diffractometer	4238 independent reflections
Radiation source: microsource	3793 reflections with *I* > 2σ(*I*)
Detector resolution: 7.41 pixels mm^-1^	*R*_int_ = 0.031
φ and ω scans	θ_max_ = 27.5°, θ_min_ = 2.9°
Absorption correction: multi-scan (*SADABS*; Krause *et al*., 2015)	*h* = −22→22
*T*_min_ = 0.919, *T*_max_ = 0.971	*k* = −10→10
30724 measured reflections	*l* = −16→17

### 4-(4-Methoxyphenyl)piperazin-1-ium 2,3,4,5,6-pentafluorobenzoate monohydrate (II) Refinement

**Table:**

Refinement on *F*^2^	Secondary atom site location: difference Fourier map
Least-squares matrix: full	Hydrogen site location: mixed
*R*[*F*^2^ > 2σ(*F*^2^)] = 0.031	H atoms treated by a mixture of independent and constrained refinement
*wR*(*F*^2^) = 0.083	*w* = 1/[σ^2^(*F*_o_^2^) + (0.0401*P*)^2^ + 0.7353*P*] where *P* = (*F*_o_^2^ + 2*F*_c_^2^)/3
*S* = 1.03	(Δ/σ)_max_ = 0.001
4238 reflections	Δρ_max_ = 0.34 e Å^−^^3^
280 parameters	Δρ_min_ = −0.18 e Å^−^^3^
0 restraints	Extinction correction: SHELXL-2019/2 (Sheldrick 2015b), Fc^*^=kFc[1+0.001xFc^2^λ^3^/sin(2θ)]^-1/4^
Primary atom site location: structure-invariant direct methods	Extinction coefficient: 0.0073 (14)

### 4-(4-Methoxyphenyl)piperazin-1-ium 2,3,4,5,6-pentafluorobenzoate monohydrate (II) Special details

**Table:**

Experimental. The crystal was mounted using polyisobutene oil on the tip of a fine glass fibre, which was fastened in a copper mounting pin with electrical solder. It was placed directly into the cold gas stream of a liquid-nitrogen based cryostat (Hope, 1994; Parkin & Hope, 1998). Diffraction data were collected with the crystal at 90K, which is standard practice in this laboratory for the majority of flash-cooled crystals.
Geometry. All esds (except the esd in the dihedral angle between two l.s. planes) are estimated using the full covariance matrix. The cell esds are taken into account individually in the estimation of esds in distances, angles and torsion angles; correlations between esds in cell parameters are only used when they are defined by crystal symmetry. An approximate (isotropic) treatment of cell esds is used for estimating esds involving l.s. planes.
Refinement. Refinement progress was checked using *Platon* (Spek, 2020) and by an *R*-tensor (Parkin, 2000). The final model was further checked with the IUCr utility *checkCIF*.

### 4-(4-Methoxyphenyl)piperazin-1-ium 2,3,4,5,6-pentafluorobenzoate monohydrate (II) Fractional atomic coordinates and isotropic or equivalent isotropic displacement parameters (Å^2^)

**Table:**

	*x*	*y*	*z*	*U*_iso_*/*U*_eq_	
N1	0.58113 (6)	0.83766 (11)	0.41244 (7)	0.01666 (19)	
H1A	0.5398 (10)	0.8859 (19)	0.3686 (12)	0.031 (4)*	
H1B	0.5880 (8)	0.8844 (17)	0.4730 (11)	0.022 (3)*	
N2	0.70053 (5)	0.59211 (11)	0.44294 (7)	0.01513 (19)	
C1	0.65689 (6)	0.85334 (13)	0.37269 (8)	0.0179 (2)	
H1C	0.672233	0.967644	0.371987	0.021*	
H1D	0.648215	0.812662	0.301405	0.021*	
C2	0.72406 (6)	0.75953 (13)	0.43956 (8)	0.0167 (2)	
H2A	0.773654	0.767857	0.411334	0.020*	
H2B	0.735304	0.804590	0.509829	0.020*	
C3	0.62849 (6)	0.57710 (13)	0.48786 (8)	0.0169 (2)	
H3A	0.639553	0.621541	0.558294	0.020*	
H3B	0.614304	0.462636	0.492007	0.020*	
C4	0.55894 (6)	0.66628 (13)	0.42271 (8)	0.0180 (2)	
H4A	0.545082	0.616403	0.353911	0.022*	
H4B	0.511236	0.659600	0.454907	0.022*	
C5	0.76277 (6)	0.48023 (13)	0.48036 (8)	0.0148 (2)	
C6	0.84193 (6)	0.52426 (13)	0.51938 (8)	0.0177 (2)	
H6	0.855236	0.634658	0.527225	0.021*	
C7	0.90243 (6)	0.40977 (14)	0.54733 (8)	0.0188 (2)	
H7	0.956319	0.442453	0.572825	0.023*	
C8	0.88343 (6)	0.24844 (13)	0.53767 (8)	0.0172 (2)	
C9	0.80383 (6)	0.20298 (13)	0.50119 (8)	0.0167 (2)	
H9	0.790398	0.092463	0.495695	0.020*	
C10	0.74434 (6)	0.31594 (13)	0.47297 (8)	0.0161 (2)	
H10	0.690445	0.282547	0.448333	0.019*	
O1	0.93660 (5)	0.1247 (1)	0.56258 (7)	0.02279 (19)	
C11	1.01917 (7)	0.16511 (16)	0.59700 (11)	0.0303 (3)	
H11A	1.051593	0.067127	0.605706	0.045*	
H11B	1.025929	0.221583	0.662767	0.045*	
H11C	1.036751	0.234384	0.546209	0.045*	
F1	0.24103 (4)	1.14509 (8)	0.27860 (5)	0.02317 (16)	
O2	0.43941 (5)	0.89873 (9)	0.27760 (6)	0.01884 (17)	
O3	0.39764 (5)	1.07117 (10)	0.38371 (6)	0.02156 (18)	
C12	0.38699 (6)	0.95869 (13)	0.32036 (8)	0.0156 (2)	
C13	0.30287 (6)	0.88933 (13)	0.29215 (8)	0.0154 (2)	
C14	0.23480 (7)	0.98503 (13)	0.27223 (8)	0.0177 (2)	
C15	0.15862 (7)	0.92190 (15)	0.24210 (8)	0.0209 (2)	
C16	0.14848 (7)	0.75875 (15)	0.23275 (8)	0.0216 (2)	
C17	0.21451 (7)	0.65934 (14)	0.25387 (8)	0.0197 (2)	
C18	0.29016 (6)	0.72513 (13)	0.28256 (8)	0.0167 (2)	
F2	0.09439 (4)	1.01803 (10)	0.22123 (6)	0.02973 (18)	
F3	0.07470 (4)	0.69754 (10)	0.20350 (6)	0.03065 (18)	
F4	0.20518 (4)	0.50048 (8)	0.24420 (6)	0.02718 (17)	
F5	0.35283 (4)	0.62368 (8)	0.30265 (5)	0.02060 (15)	
O1W	0.55228 (5)	1.20201 (11)	0.39344 (7)	0.02363 (19)	
H1W	0.5037 (11)	1.169 (2)	0.3840 (13)	0.044 (5)*	
H2W	0.5556 (10)	1.258 (2)	0.3366 (14)	0.041 (4)*	

### 4-(4-Methoxyphenyl)piperazin-1-ium 2,3,4,5,6-pentafluorobenzoate monohydrate (II) Atomic displacement parameters (Å^2^)

**Table:**

	*U*^11^	*U*^22^	*U*^33^	*U*^12^	*U*^13^	*U*^23^
N1	0.0179 (4)	0.0149 (5)	0.0171 (4)	0.0024 (3)	0.0031 (3)	−0.0009 (4)
N2	0.0149 (4)	0.0126 (4)	0.0183 (4)	0.0000 (3)	0.0044 (3)	0.0013 (3)
C1	0.0185 (5)	0.0161 (5)	0.0196 (5)	0.0011 (4)	0.0054 (4)	0.0026 (4)
C2	0.0167 (5)	0.0139 (5)	0.0196 (5)	−0.0015 (4)	0.0037 (4)	0.0011 (4)
C3	0.0175 (5)	0.0156 (5)	0.0191 (5)	0.0003 (4)	0.0068 (4)	0.0010 (4)
C4	0.0171 (5)	0.0154 (5)	0.0220 (5)	−0.0008 (4)	0.0053 (4)	−0.0015 (4)
C5	0.0170 (5)	0.0152 (5)	0.0129 (4)	0.0009 (4)	0.0044 (4)	0.0010 (4)
C6	0.0181 (5)	0.0142 (5)	0.0208 (5)	−0.0013 (4)	0.0042 (4)	0.0009 (4)
C7	0.0155 (5)	0.0187 (5)	0.0219 (5)	−0.0014 (4)	0.0026 (4)	0.0009 (4)
C8	0.0192 (5)	0.0167 (5)	0.0162 (5)	0.0035 (4)	0.0048 (4)	0.0018 (4)
C9	0.0219 (5)	0.0133 (5)	0.0148 (5)	−0.0012 (4)	0.0038 (4)	−0.0007 (4)
C10	0.0175 (5)	0.0165 (5)	0.0142 (5)	−0.0013 (4)	0.0032 (4)	−0.0009 (4)
O1	0.0184 (4)	0.0174 (4)	0.0313 (4)	0.0037 (3)	0.0018 (3)	0.0020 (3)
C11	0.0179 (6)	0.0245 (6)	0.0469 (8)	0.0051 (5)	0.0026 (5)	0.0027 (6)
F1	0.0273 (4)	0.0147 (3)	0.0285 (4)	0.0039 (3)	0.0082 (3)	0.0034 (3)
O2	0.0170 (4)	0.0195 (4)	0.0199 (4)	0.0015 (3)	0.0034 (3)	−0.0017 (3)
O3	0.0245 (4)	0.0205 (4)	0.0205 (4)	−0.0054 (3)	0.0062 (3)	−0.0059 (3)
C12	0.0187 (5)	0.0140 (5)	0.0139 (5)	−0.0003 (4)	0.0025 (4)	0.0026 (4)
C13	0.0187 (5)	0.0161 (5)	0.0117 (4)	−0.0004 (4)	0.0037 (4)	0.0007 (4)
C14	0.0228 (5)	0.0154 (5)	0.0156 (5)	0.0004 (4)	0.0057 (4)	0.0020 (4)
C15	0.0180 (5)	0.0270 (6)	0.0179 (5)	0.0041 (4)	0.0040 (4)	0.0034 (4)
C16	0.0183 (5)	0.0306 (6)	0.0160 (5)	−0.0073 (5)	0.0035 (4)	−0.0019 (4)
C17	0.0264 (6)	0.0177 (5)	0.0161 (5)	−0.0063 (4)	0.0070 (4)	−0.0030 (4)
C18	0.0209 (5)	0.0173 (5)	0.0126 (5)	0.0016 (4)	0.0048 (4)	0.0004 (4)
F2	0.0194 (3)	0.0367 (4)	0.0325 (4)	0.0090 (3)	0.0037 (3)	0.0066 (3)
F3	0.0199 (3)	0.0418 (5)	0.0297 (4)	−0.0113 (3)	0.0036 (3)	−0.0063 (3)
F4	0.0343 (4)	0.0184 (4)	0.0309 (4)	−0.0098 (3)	0.0116 (3)	−0.0072 (3)
F5	0.0246 (3)	0.0146 (3)	0.0230 (3)	0.0033 (3)	0.0056 (3)	0.0009 (2)
O1W	0.0209 (4)	0.0256 (5)	0.0233 (4)	−0.0050 (3)	0.0019 (3)	0.0035 (3)

### 4-(4-Methoxyphenyl)piperazin-1-ium 2,3,4,5,6-pentafluorobenzoate monohydrate (II) Geometric parameters (Å, º)

**Table:**

N1—C1	1.4903 (14)	C8—C9	1.3941 (15)
N1—C4	1.4932 (14)	C9—C10	1.3782 (15)
N1—H1A	0.912 (16)	C9—H9	0.9500
N1—H1B	0.882 (15)	C10—H10	0.9500
N2—C5	1.4237 (13)	O1—C11	1.4254 (14)
N2—C2	1.4573 (13)	C11—H11A	0.9800
N2—C3	1.4693 (13)	C11—H11B	0.9800
C1—C2	1.5167 (15)	C11—H11C	0.9800
C1—H1C	0.9900	F1—C14	1.3422 (13)
C1—H1D	0.9900	O2—C12	1.2519 (13)
C2—H2A	0.9900	O3—C12	1.2506 (13)
C2—H2B	0.9900	C12—C13	1.5169 (15)
C3—C4	1.5137 (15)	C13—C14	1.3863 (15)
C3—H3A	0.9900	C13—C18	1.3900 (15)
C3—H3B	0.9900	C14—C15	1.3798 (16)
C4—H4A	0.9900	C15—F2	1.3377 (13)
C4—H4B	0.9900	C15—C16	1.3758 (17)
C5—C6	1.3900 (15)	C16—F3	1.3366 (13)
C5—C10	1.4064 (15)	C16—C17	1.3780 (17)
C6—C7	1.3981 (15)	C17—F4	1.3392 (13)
C6—H6	0.9500	C17—C18	1.3788 (15)
C7—C8	1.3853 (16)	C18—F5	1.3444 (12)
C7—H7	0.9500	O1W—H1W	0.854 (19)
C8—O1	1.3686 (13)	O1W—H2W	0.899 (18)
			
C1—N1—C4	111.58 (8)	C8—C7—H7	120.2
C1—N1—H1A	110.1 (10)	C6—C7—H7	120.2
C4—N1—H1A	107.8 (10)	O1—C8—C7	125.57 (10)
C1—N1—H1B	108.6 (9)	O1—C8—C9	115.16 (10)
C4—N1—H1B	109.3 (9)	C7—C8—C9	119.25 (10)
H1A—N1—H1B	109.5 (13)	C10—C9—C8	121.0 (1)
C5—N2—C2	116.85 (8)	C10—C9—H9	119.5
C5—N2—C3	114.93 (8)	C8—C9—H9	119.5
C2—N2—C3	110.47 (8)	C9—C10—C5	120.54 (10)
N1—C1—C2	110.35 (9)	C9—C10—H10	119.7
N1—C1—H1C	109.6	C5—C10—H10	119.7
C2—C1—H1C	109.6	C8—O1—C11	117.24 (9)
N1—C1—H1D	109.6	O1—C11—H11A	109.5
C2—C1—H1D	109.6	O1—C11—H11B	109.5
H1C—C1—H1D	108.1	H11A—C11—H11B	109.5
N2—C2—C1	109.57 (9)	O1—C11—H11C	109.5
N2—C2—H2A	109.8	H11A—C11—H11C	109.5
C1—C2—H2A	109.8	H11B—C11—H11C	109.5
N2—C2—H2B	109.8	O3—C12—O2	125.87 (10)
C1—C2—H2B	109.8	O3—C12—C13	117.13 (9)
H2A—C2—H2B	108.2	O2—C12—C13	117.00 (9)
N2—C3—C4	110.04 (8)	C14—C13—C18	116.3 (1)
N2—C3—H3A	109.7	C14—C13—C12	122.31 (10)
C4—C3—H3A	109.7	C18—C13—C12	121.37 (10)
N2—C3—H3B	109.7	F1—C14—C15	117.15 (10)
C4—C3—H3B	109.7	F1—C14—C13	120.66 (10)
H3A—C3—H3B	108.2	C15—C14—C13	122.16 (10)
N1—C4—C3	109.99 (9)	F2—C15—C16	119.56 (10)
N1—C4—H4A	109.7	F2—C15—C14	120.58 (11)
C3—C4—H4A	109.7	C16—C15—C14	119.86 (11)
N1—C4—H4B	109.7	F3—C16—C15	119.88 (11)
C3—C4—H4B	109.7	F3—C16—C17	120.37 (11)
H4A—C4—H4B	108.2	C15—C16—C17	119.74 (10)
C6—C5—C10	117.98 (10)	F4—C17—C16	120.1 (1)
C6—C5—N2	123.48 (10)	F4—C17—C18	120.47 (10)
C10—C5—N2	118.47 (9)	C16—C17—C18	119.41 (11)
C5—C6—C7	121.5 (1)	F5—C18—C17	117.4 (1)
C5—C6—H6	119.2	F5—C18—C13	120.09 (10)
C7—C6—H6	119.2	C17—C18—C13	122.51 (10)
C8—C7—C6	119.69 (10)	H1W—O1W—H2W	105.2 (15)
			
C4—N1—C1—C2	−54.78 (11)	O3—C12—C13—C18	−137.95 (11)
C5—N2—C2—C1	164.82 (8)	O2—C12—C13—C18	42.46 (14)
C3—N2—C2—C1	−61.29 (11)	C18—C13—C14—F1	−179.42 (9)
N1—C1—C2—N2	57.64 (11)	C12—C13—C14—F1	−0.87 (15)
C5—N2—C3—C4	−163.83 (9)	C18—C13—C14—C15	−1.53 (15)
C2—N2—C3—C4	61.33 (11)	C12—C13—C14—C15	177.02 (10)
C1—N1—C4—C3	54.36 (11)	F1—C14—C15—F2	−0.50 (15)
N2—C3—C4—N1	−56.94 (11)	C13—C14—C15—F2	−178.46 (9)
C2—N2—C5—C6	4.50 (14)	F1—C14—C15—C16	179.1 (1)
C3—N2—C5—C6	−127.38 (11)	C13—C14—C15—C16	1.14 (16)
C2—N2—C5—C10	−172.24 (9)	F2—C15—C16—F3	−0.35 (16)
C3—N2—C5—C10	55.87 (12)	C14—C15—C16—F3	−179.95 (9)
C10—C5—C6—C7	2.19 (15)	F2—C15—C16—C17	179.80 (9)
N2—C5—C6—C7	−174.57 (10)	C14—C15—C16—C17	0.20 (17)
C5—C6—C7—C8	−1.00 (16)	F3—C16—C17—F4	0.66 (16)
C6—C7—C8—O1	−179.7 (1)	C15—C16—C17—F4	−179.49 (10)
C6—C7—C8—C9	−0.72 (16)	F3—C16—C17—C18	179.11 (9)
O1—C8—C9—C10	−179.72 (9)	C15—C16—C17—C18	−1.04 (16)
C7—C8—C9—C10	1.19 (16)	F4—C17—C18—F5	−1.69 (15)
C8—C9—C10—C5	0.04 (16)	C16—C17—C18—F5	179.86 (9)
C6—C5—C10—C9	−1.71 (15)	F4—C17—C18—C13	179.05 (10)
N2—C5—C10—C9	175.22 (9)	C16—C17—C18—C13	0.60 (16)
C7—C8—O1—C11	−3.02 (16)	C14—C13—C18—F5	−178.58 (9)
C9—C8—O1—C11	177.96 (10)	C12—C13—C18—F5	2.86 (15)
O3—C12—C13—C14	43.57 (14)	C14—C13—C18—C17	0.66 (15)
O2—C12—C13—C14	−136.02 (11)	C12—C13—C18—C17	−177.91 (9)

### 4-(4-Methoxyphenyl)piperazin-1-ium 2,3,4,5,6-pentafluorobenzoate monohydrate (II) Hydrogen-bond geometry (Å, º)

**Table:**

*D*—H···*A*	*D*—H	H···*A*	*D*···*A*	*D*—H···*A*
N1—H1*A*···O2	0.912 (16)	1.886 (16)	2.7464 (12)	156.5 (14)
N1—H1*B*···O3^i^	0.882 (15)	1.909 (15)	2.7692 (12)	164.9 (13)
C1—H1*C*···F5^ii^	0.99	2.62	3.2261 (12)	119
C3—H3*B*···O1*W*^iii^	0.99	2.65	3.5235 (14)	148
C4—H4*A*···O2^iv^	0.99	2.57	3.4812 (13)	153
C10—H10···O1*W*^iii^	0.95	2.41	3.3581 (14)	177
C11—H11*A*···F3^v^	0.98	2.55	3.3961 (15)	144
C11—H11*B*···F2^vi^	0.98	2.51	3.2436 (15)	131
O1*W*—H1*W*···O3	0.854 (19)	1.978 (19)	2.8222 (12)	170.0 (17)
O1*W*—H2*W*···O2^ii^	0.899 (18)	1.936 (19)	2.8319 (12)	173.7 (16)

Symmetry codes: (i) −*x*+1, −*y*+2, −*z*+1; (ii) −*x*+1, *y*+1/2, −*z*+1/2; (iii) *x*, *y*−1, *z*; (iv) −*x*+1, *y*−1/2, −*z*+1/2; (v) *x*+1, −*y*+1/2, *z*+1/2; (vi) *x*+1, −*y*+3/2, *z*+1/2.

### 4-(4-Methoxyphenyl)piperazin-1-ium 4-iodobenzoate monohydrate (III) Crystal data

**Table:**

C_11_H_17_N_2_O^+^·C_7_H_4_IO_2_^−^·H_2_O	*F*(000) = 2760
*M**_r_* = 458.28	*D*_x_ = 1.640 Mg m^−^^3^
Monoclinic, *P*2_1_/*c*	Mo *K*α radiation, λ = 0.71073 Å
*a* = 20.4117 (18) Å	Cell parameters from 9052 reflections
*b* = 7.4255 (6) Å	θ = 2.3–27.5°
*c* = 36.796 (3) Å	µ = 1.75 mm^−^^1^
β = 92.970 (3)°	*T* = 90 K
*V* = 5569.6 (8) Å^3^	Plate, colourless
*Z* = 12	0.30 × 0.13 × 0.03 mm

### 4-(4-Methoxyphenyl)piperazin-1-ium 4-iodobenzoate monohydrate (III) Data collection

**Table:**

Bruker D8 Venture dual source diffractometer	12876 independent reflections
Radiation source: microsource	10003 reflections with *I* > 2σ(*I*)
Detector resolution: 7.41 pixels mm^-1^	*R*_int_ = 0.056
φ and ω scans	θ_max_ = 27.7°, θ_min_ = 2.0°
Absorption correction: multi-scan (*SADABS*; Krause *et al*., 2015)	*h* = −26→26
*T*_min_ = 0.726, *T*_max_ = 0.862	*k* = −9→6
72181 measured reflections	*l* = −47→47

### 4-(4-Methoxyphenyl)piperazin-1-ium 4-iodobenzoate monohydrate (III) Refinement

**Table:**

Refinement on *F*^2^	Secondary atom site location: difference Fourier map
Least-squares matrix: full	Hydrogen site location: mixed
*R*[*F*^2^ > 2σ(*F*^2^)] = 0.033	H atoms treated by a mixture of independent and constrained refinement
*wR*(*F*^2^) = 0.069	*w* = 1/[σ^2^(*F*_o_^2^) + (0.0216*P*)^2^ + 1.7628*P*] where *P* = (*F*_o_^2^ + 2*F*_c_^2^)/3
*S* = 1.03	(Δ/σ)_max_ = 0.002
12876 reflections	Δρ_max_ = 0.84 e Å^−^^3^
729 parameters	Δρ_min_ = −0.80 e Å^−^^3^
12 restraints	Extinction correction: SHELXL-2019/2 (Sheldrick 2015b), Fc^*^=kFc[1+0.001xFc^2^λ^3^/sin(2θ)]^-1/4^
Primary atom site location: structure-invariant direct methods	Extinction coefficient: 0.00017 (3)

### 4-(4-Methoxyphenyl)piperazin-1-ium 4-iodobenzoate monohydrate (III) Special details

**Table:**

Experimental. The crystal was mounted using polyisobutene oil on the tip of a fine glass fibre, which was fastened in a copper mounting pin with electrical solder. It was placed directly into the cold gas stream of a liquid-nitrogen based cryostat (Hope, 1994; Parkin & Hope, 1998). Diffraction data were collected with the crystal at 90K, which is standard practice in this laboratory for the majority of flash-cooled crystals.
Geometry. All esds (except the esd in the dihedral angle between two l.s. planes) are estimated using the full covariance matrix. The cell esds are taken into account individually in the estimation of esds in distances, angles and torsion angles; correlations between esds in cell parameters are only used when they are defined by crystal symmetry. An approximate (isotropic) treatment of cell esds is used for estimating esds involving l.s. planes.
Refinement. Refinement progress was checked using *Platon* (Spek, 2020) and by an *R*-tensor (Parkin, 2000). The final model was further checked with the IUCr utility *checkCIF*.

### 4-(4-Methoxyphenyl)piperazin-1-ium 4-iodobenzoate monohydrate (III) Fractional atomic coordinates and isotropic or equivalent isotropic displacement parameters (Å^2^)

**Table:**

	*x*	*y*	*z*	*U*_iso_*/*U*_eq_	
O1W	0.43969 (10)	0.2205 (3)	0.70712 (5)	0.0221 (4)	
H1W1	0.4236 (15)	0.181 (4)	0.7238 (6)	0.043 (10)*	
H2W1	0.4260 (15)	0.166 (4)	0.6906 (6)	0.043 (10)*	
O2W	0.46521 (10)	0.3419 (3)	0.54225 (5)	0.0203 (4)	
H1W2	0.4460 (12)	0.310 (4)	0.5585 (5)	0.024 (8)*	
H2W2	0.4469 (14)	0.300 (4)	0.5253 (5)	0.046 (10)*	
O3W	0.47153 (9)	0.4637 (3)	0.38524 (5)	0.0189 (4)	
H1W3	0.4493 (13)	0.439 (4)	0.4008 (6)	0.039 (9)*	
H2W3	0.4552 (13)	0.417 (4)	0.3683 (5)	0.031 (9)*	
N1A	0.53728 (11)	0.1701 (3)	0.62582 (6)	0.0169 (5)	
H1AA	0.4979 (16)	0.123 (4)	0.6303 (8)	0.039 (9)*	
H1AB	0.5305 (15)	0.293 (4)	0.6212 (8)	0.036 (9)*	
N2A	0.67722 (10)	0.1578 (3)	0.61970 (5)	0.0153 (5)	
C1A	0.56652 (12)	0.0802 (3)	0.59424 (7)	0.0166 (6)	
H1AC	0.536526	0.091693	0.572342	0.020*	
H1AD	0.573059	−0.049571	0.599461	0.020*	
C2A	0.63171 (12)	0.1678 (3)	0.58741 (6)	0.0155 (5)	
H2AA	0.651615	0.106930	0.566729	0.019*	
H2AB	0.624385	0.295573	0.580691	0.019*	
C3A	0.64855 (13)	0.2351 (4)	0.65186 (6)	0.0173 (6)	
H3A1	0.643210	0.366608	0.648468	0.021*	
H3A2	0.678752	0.215151	0.673412	0.021*	
C4A	0.58240 (13)	0.1516 (4)	0.65859 (7)	0.0190 (6)	
H4AA	0.588228	0.022477	0.664660	0.023*	
H4AB	0.563121	0.211922	0.679520	0.023*	
C5A	0.74157 (13)	0.2193 (3)	0.61344 (6)	0.0150 (5)	
C6A	0.77415 (13)	0.1533 (3)	0.58374 (7)	0.0175 (6)	
H6A	0.752173	0.070253	0.567695	0.021*	
C7A	0.83727 (13)	0.2051 (3)	0.57702 (7)	0.0186 (6)	
H7A	0.857901	0.158525	0.556458	0.022*	
C8A	0.87077 (13)	0.3250 (3)	0.60019 (7)	0.0172 (6)	
C9A	0.83902 (13)	0.3916 (4)	0.62985 (7)	0.0187 (6)	
H9A	0.861272	0.473954	0.645926	0.022*	
C10A	0.77587 (13)	0.3404 (3)	0.63637 (7)	0.0181 (6)	
H10A	0.755274	0.388262	0.656829	0.022*	
O1A	0.93324 (9)	0.3865 (3)	0.59612 (5)	0.0246 (5)	
C11A	0.96791 (14)	0.3085 (4)	0.56715 (7)	0.0277 (7)	
H11A	1.012352	0.358505	0.567375	0.042*	
H11B	0.944723	0.335895	0.543819	0.042*	
H11C	0.970366	0.177628	0.570434	0.042*	
I1A	0.07054 (2)	0.22414 (3)	0.65985 (2)	0.03241 (6)	
O2A	0.41019 (9)	0.0741 (2)	0.64110 (4)	0.0182 (4)	
O3A	0.38729 (10)	0.2563 (3)	0.59416 (5)	0.0327 (5)	
C12A	0.37115 (13)	0.1708 (3)	0.62136 (7)	0.0177 (6)	
C13A	0.30062 (13)	0.1839 (3)	0.63110 (6)	0.0155 (6)	
C14A	0.25598 (14)	0.2798 (3)	0.60871 (7)	0.0202 (6)	
H14A	0.270779	0.337633	0.587618	0.024*	
C15A	0.19067 (14)	0.2927 (4)	0.61649 (7)	0.0235 (6)	
H15A	0.160641	0.357675	0.600863	0.028*	
C16A	0.16966 (13)	0.2086 (3)	0.64770 (7)	0.0189 (6)	
C17A	0.21308 (13)	0.1131 (3)	0.67042 (7)	0.0185 (6)	
H17A	0.198222	0.056099	0.691601	0.022*	
C18A	0.27842 (13)	0.1008 (3)	0.66215 (6)	0.0150 (5)	
H18A	0.308316	0.035272	0.677757	0.018*	
N1B	0.54528 (11)	0.2872 (3)	0.46279 (6)	0.0157 (5)	
H1BA	0.5047 (15)	0.247 (4)	0.4678 (8)	0.032 (9)*	
H1BB	0.5413 (13)	0.410 (4)	0.4599 (7)	0.019 (7)*	
N2B	0.68341 (10)	0.2390 (3)	0.45357 (5)	0.0145 (5)	
C1B	0.57009 (12)	0.1948 (3)	0.43022 (6)	0.0156 (6)	
H1BC	0.539379	0.215359	0.408906	0.019*	
H1BD	0.572919	0.063524	0.434700	0.019*	
C2B	0.63691 (12)	0.2675 (3)	0.42252 (6)	0.0151 (5)	
H2BA	0.653183	0.206538	0.400822	0.018*	
H2BB	0.633515	0.397882	0.417175	0.018*	
C3B	0.66000 (12)	0.3265 (3)	0.48621 (6)	0.0161 (6)	
H3B1	0.658596	0.458500	0.482408	0.019*	
H3B2	0.691065	0.301479	0.507155	0.019*	
C4B	0.59236 (13)	0.2603 (4)	0.49477 (6)	0.0173 (6)	
H4BA	0.594522	0.130750	0.501113	0.021*	
H4BB	0.576808	0.326780	0.515985	0.021*	
C5B	0.75017 (12)	0.2699 (3)	0.44638 (6)	0.0134 (5)	
C6B	0.77627 (13)	0.1969 (3)	0.41512 (7)	0.0166 (6)	
H6B	0.747807	0.135704	0.398002	0.020*	
C7B	0.84192 (13)	0.2109 (3)	0.40838 (7)	0.0181 (6)	
H7B	0.857875	0.160425	0.386830	0.022*	
C8B	0.88469 (13)	0.2985 (3)	0.43297 (7)	0.0185 (6)	
C9B	0.85954 (13)	0.3744 (4)	0.46404 (7)	0.0208 (6)	
H9B	0.888096	0.435936	0.481045	0.025*	
C10B	0.79355 (13)	0.3610 (3)	0.47032 (7)	0.0182 (6)	
H10B	0.777431	0.415177	0.491472	0.022*	
O1B	0.95052 (9)	0.3195 (3)	0.42936 (5)	0.0280 (5)	
C11B	0.97811 (14)	0.2421 (4)	0.39805 (8)	0.0303 (7)	
H11D	1.025183	0.268615	0.398486	0.045*	
H11E	0.956558	0.293296	0.375985	0.045*	
H11F	0.971528	0.111410	0.398221	0.045*	
I1B	0.07478 (2)	0.15453 (3)	0.49437 (2)	0.02872 (6)	
O2B	0.41837 (9)	0.1894 (2)	0.47849 (5)	0.0198 (4)	
O3B	0.38573 (10)	0.3743 (3)	0.43324 (5)	0.0298 (5)	
C12B	0.37472 (13)	0.2739 (3)	0.45938 (7)	0.0178 (6)	
C13B	0.30402 (13)	0.2499 (3)	0.46834 (6)	0.0145 (5)	
C14B	0.25389 (13)	0.3216 (3)	0.44580 (7)	0.0179 (6)	
H14B	0.264596	0.389536	0.425054	0.022*	
C15B	0.18860 (14)	0.2958 (4)	0.45298 (7)	0.0201 (6)	
H15B	0.154697	0.343937	0.437190	0.024*	
C16B	0.17363 (13)	0.1985 (3)	0.48365 (7)	0.0186 (6)	
C17B	0.22246 (13)	0.1289 (3)	0.50692 (7)	0.0186 (6)	
H17B	0.211464	0.064335	0.528047	0.022*	
C18B	0.28755 (13)	0.1538 (3)	0.49930 (6)	0.0163 (6)	
H18B	0.321254	0.105354	0.515187	0.020*	
N1C	0.54732 (11)	0.4096 (3)	0.30188 (6)	0.0151 (5)	
H1CA	0.5039 (15)	0.382 (4)	0.3092 (8)	0.036 (9)*	
H1CB	0.5487 (13)	0.519 (4)	0.2976 (7)	0.012 (7)*	
N2C	0.67789 (10)	0.2903 (3)	0.28952 (5)	0.0140 (5)	
C1C	0.56203 (13)	0.3023 (4)	0.26914 (7)	0.0183 (6)	
H1CC	0.531549	0.337052	0.248527	0.022*	
H1CD	0.555525	0.172679	0.274144	0.022*	
C2C	0.63187 (12)	0.3341 (4)	0.25908 (6)	0.0168 (6)	
H2CA	0.641494	0.258599	0.237828	0.020*	
H2CB	0.637343	0.461852	0.252166	0.020*	
C3C	0.66487 (13)	0.3992 (4)	0.32146 (6)	0.0164 (6)	
H3C1	0.671354	0.528155	0.315826	0.020*	
H3C2	0.696146	0.366146	0.341838	0.020*	
C4C	0.59550 (12)	0.3697 (4)	0.33264 (6)	0.0164 (6)	
H4CA	0.590283	0.243214	0.340487	0.020*	
H4CB	0.586798	0.448762	0.353511	0.020*	
C5C	0.74472 (12)	0.2758 (3)	0.28062 (6)	0.0141 (5)	
C6C	0.76069 (13)	0.1706 (3)	0.25095 (7)	0.0177 (6)	
H6C	0.726496	0.118494	0.235930	0.021*	
C7C	0.82550 (13)	0.1404 (4)	0.24290 (7)	0.0186 (6)	
H7C	0.835037	0.070293	0.222230	0.022*	
C8C	0.87656 (13)	0.2121 (4)	0.26487 (7)	0.0196 (6)	
C9C	0.86090 (13)	0.3184 (4)	0.29422 (7)	0.0204 (6)	
H9C	0.895187	0.369408	0.309346	0.025*	
C10C	0.79633 (13)	0.3517 (3)	0.30189 (7)	0.0177 (6)	
H10C	0.786977	0.427006	0.321839	0.021*	
O1C	0.94180 (9)	0.1861 (3)	0.26005 (5)	0.0250 (4)	
C11C	0.95839 (14)	0.0599 (4)	0.23304 (8)	0.0314 (7)	
H11G	1.006183	0.046967	0.233207	0.047*	
H11H	0.941860	0.102414	0.209073	0.047*	
H11I	0.938558	−0.056985	0.238193	0.047*	
I1C	0.09042 (2)	0.06781 (3)	0.31910 (2)	0.03020 (6)	
O2C	0.42707 (9)	0.3016 (2)	0.32290 (5)	0.0202 (4)	
O3C	0.39400 (9)	0.4141 (2)	0.26896 (5)	0.0235 (4)	
C12C	0.38327 (13)	0.3315 (3)	0.29758 (7)	0.0167 (6)	
C13C	0.31525 (13)	0.2627 (3)	0.30286 (7)	0.0155 (6)	
C14C	0.27202 (13)	0.2325 (4)	0.27301 (7)	0.0192 (6)	
H14C	0.286224	0.254050	0.249216	0.023*	
C15C	0.20884 (14)	0.1719 (4)	0.27722 (7)	0.0212 (6)	
H15C	0.180102	0.148483	0.256620	0.025*	
C16C	0.18836 (13)	0.1459 (3)	0.31218 (7)	0.0198 (6)	
C17C	0.23004 (13)	0.1737 (3)	0.34227 (7)	0.0204 (6)	
H17C	0.215262	0.154375	0.366023	0.024*	
C18C	0.29359 (13)	0.2299 (3)	0.33759 (7)	0.0172 (6)	
H18C	0.322853	0.246385	0.358253	0.021*	

### 4-(4-Methoxyphenyl)piperazin-1-ium 4-iodobenzoate monohydrate (III) Atomic displacement parameters (Å^2^)

**Table:**

	*U*^11^	*U*^22^	*U*^33^	*U*^12^	*U*^13^	*U*^23^
O1W	0.0265 (12)	0.0229 (11)	0.0167 (10)	−0.0040 (9)	−0.0009 (9)	−0.0004 (9)
O2W	0.0214 (11)	0.0235 (11)	0.0165 (10)	−0.0031 (9)	0.0052 (9)	0.0010 (9)
O3W	0.0203 (11)	0.0206 (11)	0.0162 (10)	−0.0003 (9)	0.0046 (9)	−0.0003 (9)
N1A	0.0159 (13)	0.0171 (12)	0.0181 (11)	−0.0011 (10)	0.0039 (9)	0.0005 (10)
N2A	0.0131 (11)	0.0181 (11)	0.0149 (10)	−0.0004 (9)	0.0023 (9)	−0.0008 (9)
C1A	0.0151 (14)	0.0154 (13)	0.0195 (13)	0.0004 (11)	0.0023 (11)	−0.0022 (11)
C2A	0.0143 (14)	0.0174 (13)	0.0148 (12)	−0.0001 (11)	0.0021 (10)	−0.0003 (11)
C3A	0.0176 (14)	0.0226 (14)	0.0118 (12)	0.0011 (12)	0.0027 (10)	−0.0008 (11)
C4A	0.0172 (14)	0.0234 (15)	0.0169 (13)	0.0007 (12)	0.0045 (11)	0.0036 (11)
C5A	0.0144 (14)	0.0156 (13)	0.0151 (12)	0.0021 (11)	0.002 (1)	0.002 (1)
C6A	0.0169 (14)	0.0176 (14)	0.0178 (13)	−0.0018 (11)	0.0005 (11)	−0.0041 (11)
C7A	0.0190 (15)	0.0186 (14)	0.0184 (13)	−0.0003 (12)	0.0037 (11)	−0.0044 (11)
C8A	0.0135 (14)	0.0154 (13)	0.0227 (13)	0.0011 (11)	0.0023 (11)	0.0019 (11)
C9A	0.0188 (15)	0.0187 (14)	0.0184 (13)	−0.0014 (12)	−0.0022 (11)	−0.0046 (11)
C10A	0.0192 (15)	0.0209 (14)	0.0144 (12)	0.0023 (12)	0.0023 (11)	−0.0048 (11)
O1A	0.0158 (10)	0.0288 (11)	0.0298 (11)	−0.0064 (9)	0.0071 (8)	−0.0099 (9)
C11A	0.0202 (16)	0.0354 (18)	0.0285 (15)	−0.0036 (14)	0.0106 (13)	−0.0057 (13)
I1A	0.01674 (11)	0.02892 (11)	0.05171 (13)	0.00099 (9)	0.00330 (9)	−0.01156 (10)
O2A	0.0175 (10)	0.0203 (10)	0.0172 (9)	−0.0023 (8)	0.0037 (8)	−0.0022 (8)
O3A	0.0343 (13)	0.0381 (13)	0.0272 (11)	−0.0018 (10)	0.0153 (10)	0.0115 (9)
C12A	0.0219 (15)	0.0157 (13)	0.0161 (13)	−0.0048 (12)	0.0049 (11)	−0.0050 (11)
C13A	0.0187 (14)	0.0117 (13)	0.0163 (12)	−0.0049 (11)	0.0036 (11)	−0.0033 (10)
C14A	0.0309 (17)	0.0133 (13)	0.0165 (13)	−0.0043 (12)	0.0016 (12)	0.0018 (11)
C15A	0.0240 (16)	0.0169 (14)	0.0289 (15)	0.0013 (12)	−0.0063 (13)	−0.0009 (12)
C16A	0.0144 (14)	0.0157 (13)	0.0266 (14)	−0.0033 (11)	0.0008 (11)	−0.0078 (11)
C17A	0.0194 (15)	0.0163 (13)	0.0202 (13)	−0.0021 (12)	0.0054 (11)	0.0001 (11)
C18A	0.0183 (14)	0.0118 (12)	0.0150 (12)	−0.0017 (11)	0.0014 (10)	−0.0003 (10)
N1B	0.0149 (12)	0.0150 (12)	0.0173 (11)	−0.0009 (10)	0.0026 (9)	−0.0006 (9)
N2B	0.0153 (12)	0.0160 (11)	0.0122 (10)	−0.0009 (9)	0.0012 (9)	−0.0021 (9)
C1B	0.0146 (14)	0.0169 (13)	0.0156 (12)	−0.0003 (11)	0.0018 (10)	−0.0011 (10)
C2B	0.0160 (14)	0.0181 (13)	0.0112 (12)	0.0007 (11)	0.0017 (10)	−0.0018 (10)
C3B	0.0172 (14)	0.0200 (14)	0.0113 (12)	−0.0013 (11)	0.0015 (10)	−0.002 (1)
C4B	0.0186 (14)	0.0197 (14)	0.0137 (12)	−0.0005 (12)	0.0018 (11)	0.0003 (11)
C5B	0.0164 (14)	0.0098 (12)	0.0137 (12)	0.0006 (11)	−0.0013 (10)	0.0015 (10)
C6B	0.0187 (14)	0.0145 (13)	0.0163 (13)	−0.0023 (11)	−0.0005 (11)	−0.0029 (10)
C7B	0.0201 (15)	0.0158 (13)	0.0186 (13)	−0.0015 (12)	0.0026 (11)	−0.0044 (11)
C8B	0.0141 (14)	0.0169 (14)	0.0247 (14)	0.0006 (11)	0.0020 (11)	0.0000 (11)
C9B	0.0206 (15)	0.0222 (15)	0.0193 (13)	−0.0040 (12)	−0.0032 (11)	−0.0065 (11)
C10B	0.0218 (15)	0.0182 (14)	0.0147 (12)	0.0024 (12)	0.0019 (11)	−0.0032 (11)
O1B	0.0137 (10)	0.0354 (12)	0.0351 (11)	−0.0036 (9)	0.0042 (9)	−0.0124 (9)
C11B	0.0209 (16)	0.0357 (18)	0.0352 (17)	−0.0038 (14)	0.0105 (13)	−0.0099 (14)
I1B	0.01675 (10)	0.02811 (11)	0.04205 (12)	−0.00272 (8)	0.00875 (8)	−0.00998 (9)
O2B	0.0172 (10)	0.0225 (10)	0.0201 (9)	−0.0013 (8)	0.0046 (8)	−0.0032 (8)
O3B	0.0288 (12)	0.0302 (12)	0.0318 (11)	−0.0017 (10)	0.0138 (9)	0.0118 (9)
C12B	0.0227 (15)	0.0143 (13)	0.0168 (13)	−0.0043 (12)	0.0061 (11)	−0.0069 (11)
C13B	0.0195 (14)	0.0099 (12)	0.0142 (12)	−0.0023 (11)	0.0042 (11)	−0.0035 (10)
C14B	0.0244 (15)	0.0155 (13)	0.0139 (12)	−0.0051 (12)	0.0003 (11)	−0.0001 (10)
C15B	0.0212 (15)	0.0175 (14)	0.0212 (14)	−0.0001 (12)	−0.0036 (11)	−0.0016 (11)
C16B	0.0151 (14)	0.0159 (13)	0.0254 (14)	−0.0006 (11)	0.0063 (11)	−0.0054 (11)
C17B	0.0226 (15)	0.0139 (13)	0.0200 (13)	−0.0004 (12)	0.0078 (11)	0.0017 (11)
C18B	0.0193 (14)	0.0153 (13)	0.0143 (12)	0.0017 (11)	0.0020 (11)	0.0010 (11)
N1C	0.0162 (13)	0.0145 (12)	0.0147 (11)	−0.0014 (10)	0.0001 (9)	0.0002 (9)
N2C	0.0143 (11)	0.0166 (11)	0.0111 (10)	0.0006 (9)	0.0010 (9)	−0.0015 (8)
C1C	0.0172 (14)	0.0209 (14)	0.0169 (13)	0.0001 (12)	0.0005 (11)	−0.0040 (11)
C2C	0.0158 (14)	0.0212 (14)	0.0132 (12)	0.0010 (11)	−0.0005 (10)	−0.0002 (11)
C3C	0.0177 (14)	0.0198 (14)	0.0116 (12)	0.0000 (11)	0.0003 (10)	−0.0012 (10)
C4C	0.0195 (14)	0.0181 (14)	0.0116 (12)	0.0016 (12)	0.0002 (10)	0.0009 (10)
C5C	0.0149 (14)	0.0127 (12)	0.0146 (12)	−0.0019 (11)	0.0001 (10)	0.0027 (10)
C6C	0.0163 (14)	0.0201 (14)	0.0166 (13)	−0.0025 (12)	−0.0015 (11)	−0.0024 (11)
C7C	0.0173 (14)	0.0208 (14)	0.0182 (13)	0.0001 (12)	0.0038 (11)	−0.0027 (11)
C8C	0.0181 (15)	0.0165 (13)	0.0245 (14)	−0.0009 (12)	0.0045 (12)	0.0057 (11)
C9C	0.0190 (15)	0.0197 (14)	0.0222 (14)	−0.0047 (12)	−0.0031 (11)	−0.0014 (11)
C10C	0.0213 (15)	0.0135 (13)	0.0183 (13)	−0.0012 (12)	0.0006 (11)	−0.0013 (11)
O1C	0.0143 (10)	0.0282 (11)	0.0330 (11)	−0.0004 (9)	0.0054 (8)	−0.0037 (9)
C11C	0.0221 (17)	0.0376 (18)	0.0352 (17)	0.0049 (14)	0.0083 (13)	−0.0043 (15)
I1C	0.01743 (10)	0.02945 (11)	0.04390 (12)	−0.00327 (8)	0.00347 (8)	−0.00136 (9)
O2C	0.0165 (10)	0.0249 (11)	0.0194 (9)	−0.0022 (8)	0.0017 (8)	0.0013 (8)
O3C	0.0266 (11)	0.0239 (10)	0.0208 (10)	0.0009 (9)	0.0075 (8)	0.0070 (8)
C12C	0.0204 (15)	0.0119 (13)	0.0184 (13)	0.0016 (11)	0.0061 (11)	−0.0043 (11)
C13C	0.0203 (15)	0.0090 (12)	0.0175 (13)	0.0026 (11)	0.0026 (11)	−0.0012 (10)
C14C	0.0201 (15)	0.0206 (14)	0.0172 (13)	0.0042 (12)	0.0030 (11)	−0.0018 (11)
C15C	0.0216 (15)	0.0200 (14)	0.0215 (14)	0.0033 (12)	−0.0030 (12)	−0.0060 (12)
C16C	0.0134 (14)	0.0149 (13)	0.0311 (15)	0.0027 (11)	0.0031 (12)	−0.0010 (12)
C17C	0.0224 (15)	0.0179 (14)	0.0213 (13)	0.0026 (12)	0.0050 (12)	0.0033 (11)
C18C	0.0188 (15)	0.0160 (13)	0.0168 (13)	0.0009 (11)	0.0001 (11)	0.0013 (11)

### 4-(4-Methoxyphenyl)piperazin-1-ium 4-iodobenzoate monohydrate (III) Geometric parameters (Å, º)

**Table:**

O1W—H1W1	0.769 (15)	C6B—H6B	0.9500
O1W—H2W1	0.768 (15)	C7B—C8B	1.386 (4)
O2W—H1W2	0.770 (14)	C7B—H7B	0.9500
O2W—H2W2	0.774 (15)	C8B—O1B	1.366 (3)
O3W—H1W3	0.771 (15)	C8B—C9B	1.396 (3)
O3W—H2W3	0.775 (14)	C9B—C10B	1.382 (4)
N1A—C4A	1.486 (3)	C9B—H9B	0.9500
N1A—C1A	1.491 (3)	C10B—H10B	0.9500
N1A—H1AA	0.90 (3)	O1B—C11B	1.429 (3)
N1A—H1AB	0.94 (3)	C11B—H11D	0.9800
N2A—C5A	1.421 (3)	C11B—H11E	0.9800
N2A—C3A	1.464 (3)	C11B—H11F	0.9800
N2A—C2A	1.472 (3)	I1B—C16B	2.101 (3)
C1A—C2A	1.514 (3)	O2B—C12B	1.272 (3)
C1A—H1AC	0.9900	O3B—C12B	1.246 (3)
C1A—H1AD	0.9900	C12B—C13B	1.507 (3)
C2A—H2AA	0.9900	C13B—C14B	1.389 (4)
C2A—H2AB	0.9900	C13B—C18B	1.400 (3)
C3A—C4A	1.518 (3)	C14B—C15B	1.385 (4)
C3A—H3A1	0.9900	C14B—H14B	0.9500
C3A—H3A2	0.9900	C15B—C16B	1.388 (4)
C4A—H4AA	0.9900	C15B—H15B	0.9500
C4A—H4AB	0.9900	C16B—C17B	1.380 (4)
C5A—C10A	1.396 (4)	C17B—C18B	1.384 (3)
C5A—C6A	1.397 (3)	C17B—H17B	0.9500
C6A—C7A	1.379 (3)	C18B—H18B	0.9500
C6A—H6A	0.9500	N1C—C1C	1.488 (3)
C7A—C8A	1.388 (4)	N1C—C4C	1.490 (3)
C7A—H7A	0.9500	N1C—H1CA	0.96 (3)
C8A—O1A	1.370 (3)	N1C—H1CB	0.83 (3)
C8A—C9A	1.389 (3)	N2C—C5C	1.424 (3)
C9A—C10A	1.377 (4)	N2C—C2C	1.461 (3)
C9A—H9A	0.9500	N2C—C3C	1.462 (3)
C10A—H10A	0.9500	C1C—C2C	1.510 (3)
O1A—C11A	1.432 (3)	C1C—H1CC	0.9900
C11A—H11A	0.9800	C1C—H1CD	0.9900
C11A—H11B	0.9800	C2C—H2CA	0.9900
C11A—H11C	0.9800	C2C—H2CB	0.9900
I1A—C16A	2.098 (3)	C3C—C4C	1.511 (3)
O2A—C12A	1.272 (3)	C3C—H3C1	0.9900
O3A—C12A	1.244 (3)	C3C—H3C2	0.9900
C12A—C13A	1.505 (3)	C4C—H4CA	0.9900
C13A—C14A	1.392 (4)	C4C—H4CB	0.9900
C13A—C18A	1.394 (3)	C5C—C6C	1.395 (3)
C14A—C15A	1.381 (4)	C5C—C10C	1.398 (4)
C14A—H14A	0.9500	C6C—C7C	1.389 (3)
C15A—C16A	1.394 (4)	C6C—H6C	0.9500
C15A—H15A	0.9500	C7C—C8C	1.392 (4)
C16A—C17A	1.383 (4)	C7C—H7C	0.9500
C17A—C18A	1.386 (3)	C8C—O1C	1.366 (3)
C17A—H17A	0.9500	C8C—C9C	1.388 (4)
C18A—H18A	0.9500	C9C—C10C	1.384 (4)
N1B—C1B	1.492 (3)	C9C—H9C	0.9500
N1B—C4B	1.494 (3)	C10C—H10C	0.9500
N1B—H1BA	0.91 (3)	O1C—C11C	1.420 (3)
N1B—H1BB	0.92 (3)	C11C—H11G	0.9800
N2B—C5B	1.420 (3)	C11C—H11H	0.9800
N2B—C2B	1.462 (3)	C11C—H11I	0.9800
N2B—C3B	1.467 (3)	I1C—C16C	2.110 (3)
C1B—C2B	1.507 (3)	O2C—C12C	1.277 (3)
C1B—H1BC	0.9900	O3C—C12C	1.248 (3)
C1B—H1BD	0.9900	C12C—C13C	1.501 (4)
C2B—H2BA	0.9900	C13C—C14C	1.391 (4)
C2B—H2BB	0.9900	C13C—C18C	1.395 (3)
C3B—C4B	1.514 (3)	C14C—C15C	1.382 (4)
C3B—H3B1	0.9900	C14C—H14C	0.9500
C3B—H3B2	0.9900	C15C—C16C	1.387 (3)
C4B—H4BA	0.9900	C15C—H15C	0.9500
C4B—H4BB	0.9900	C16C—C17C	1.376 (4)
C5B—C10B	1.392 (4)	C17C—C18C	1.382 (4)
C5B—C6B	1.402 (3)	C17C—H17C	0.9500
C6B—C7B	1.379 (3)	C18C—H18C	0.9500
			
H1W1—O1W—H2W1	106 (2)	C5B—C6B—H6B	118.9
H1W2—O2W—H2W2	105 (2)	C6B—C7B—C8B	120.2 (2)
H1W3—O3W—H2W3	104 (2)	C6B—C7B—H7B	119.9
C4A—N1A—C1A	109.6 (2)	C8B—C7B—H7B	119.9
C4A—N1A—H1AA	110 (2)	O1B—C8B—C7B	125.4 (2)
C1A—N1A—H1AA	111 (2)	O1B—C8B—C9B	116.0 (2)
C4A—N1A—H1AB	108.4 (19)	C7B—C8B—C9B	118.6 (2)
C1A—N1A—H1AB	110.9 (18)	C10B—C9B—C8B	120.7 (2)
H1AA—N1A—H1AB	107 (3)	C10B—C9B—H9B	119.6
C5A—N2A—C3A	114.6 (2)	C8B—C9B—H9B	119.6
C5A—N2A—C2A	113.78 (19)	C9B—C10B—C5B	121.5 (2)
C3A—N2A—C2A	111.8 (2)	C9B—C10B—H10B	119.2
N1A—C1A—C2A	109.1 (2)	C5B—C10B—H10B	119.2
N1A—C1A—H1AC	109.9	C8B—O1B—C11B	117.6 (2)
C2A—C1A—H1AC	109.9	O1B—C11B—H11D	109.5
N1A—C1A—H1AD	109.9	O1B—C11B—H11E	109.5
C2A—C1A—H1AD	109.9	H11D—C11B—H11E	109.5
H1AC—C1A—H1AD	108.3	O1B—C11B—H11F	109.5
N2A—C2A—C1A	111.6 (2)	H11D—C11B—H11F	109.5
N2A—C2A—H2AA	109.3	H11E—C11B—H11F	109.5
C1A—C2A—H2AA	109.3	O3B—C12B—O2B	124.9 (2)
N2A—C2A—H2AB	109.3	O3B—C12B—C13B	116.9 (2)
C1A—C2A—H2AB	109.3	O2B—C12B—C13B	118.1 (2)
H2AA—C2A—H2AB	108.0	C14B—C13B—C18B	118.7 (2)
N2A—C3A—C4A	111.6 (2)	C14B—C13B—C12B	120.5 (2)
N2A—C3A—H3A1	109.3	C18B—C13B—C12B	120.8 (2)
C4A—C3A—H3A1	109.3	C15B—C14B—C13B	121.3 (2)
N2A—C3A—H3A2	109.3	C15B—C14B—H14B	119.4
C4A—C3A—H3A2	109.3	C13B—C14B—H14B	119.4
H3A1—C3A—H3A2	108.0	C14B—C15B—C16B	118.8 (3)
N1A—C4A—C3A	110.4 (2)	C14B—C15B—H15B	120.6
N1A—C4A—H4AA	109.6	C16B—C15B—H15B	120.6
C3A—C4A—H4AA	109.6	C17B—C16B—C15B	121.1 (2)
N1A—C4A—H4AB	109.6	C17B—C16B—I1B	119.67 (18)
C3A—C4A—H4AB	109.6	C15B—C16B—I1B	119.2 (2)
H4AA—C4A—H4AB	108.1	C16B—C17B—C18B	119.6 (2)
C10A—C5A—C6A	117.0 (2)	C16B—C17B—H17B	120.2
C10A—C5A—N2A	123.2 (2)	C18B—C17B—H17B	120.2
C6A—C5A—N2A	119.7 (2)	C17B—C18B—C13B	120.4 (3)
C7A—C6A—C5A	122.0 (2)	C17B—C18B—H18B	119.8
C7A—C6A—H6A	119.0	C13B—C18B—H18B	119.8
C5A—C6A—H6A	119.0	C1C—N1C—C4C	110.8 (2)
C6A—C7A—C8A	120.3 (2)	C1C—N1C—H1CA	110.1 (18)
C6A—C7A—H7A	119.9	C4C—N1C—H1CA	109.0 (18)
C8A—C7A—H7A	119.9	C1C—N1C—H1CB	111.1 (17)
O1A—C8A—C7A	125.3 (2)	C4C—N1C—H1CB	108.2 (19)
O1A—C8A—C9A	116.2 (2)	H1CA—N1C—H1CB	108 (3)
C7A—C8A—C9A	118.4 (2)	C5C—N2C—C2C	115.22 (19)
C10A—C9A—C8A	121.2 (2)	C5C—N2C—C3C	116.3 (2)
C10A—C9A—H9A	119.4	C2C—N2C—C3C	110.9 (2)
C8A—C9A—H9A	119.4	N1C—C1C—C2C	110.3 (2)
C9A—C10A—C5A	121.1 (2)	N1C—C1C—H1CC	109.6
C9A—C10A—H10A	119.4	C2C—C1C—H1CC	109.6
C5A—C10A—H10A	119.4	N1C—C1C—H1CD	109.6
C8A—O1A—C11A	116.4 (2)	C2C—C1C—H1CD	109.6
O1A—C11A—H11A	109.5	H1CC—C1C—H1CD	108.1
O1A—C11A—H11B	109.5	N2C—C2C—C1C	110.8 (2)
H11A—C11A—H11B	109.5	N2C—C2C—H2CA	109.5
O1A—C11A—H11C	109.5	C1C—C2C—H2CA	109.5
H11A—C11A—H11C	109.5	N2C—C2C—H2CB	109.5
H11B—C11A—H11C	109.5	C1C—C2C—H2CB	109.5
O3A—C12A—O2A	124.3 (2)	H2CA—C2C—H2CB	108.1
O3A—C12A—C13A	117.1 (2)	N2C—C3C—C4C	110.5 (2)
O2A—C12A—C13A	118.6 (2)	N2C—C3C—H3C1	109.5
C14A—C13A—C18A	118.7 (2)	C4C—C3C—H3C1	109.5
C14A—C13A—C12A	119.8 (2)	N2C—C3C—H3C2	109.5
C18A—C13A—C12A	121.5 (2)	C4C—C3C—H3C2	109.5
C15A—C14A—C13A	121.5 (2)	H3C1—C3C—H3C2	108.1
C15A—C14A—H14A	119.3	N1C—C4C—C3C	110.8 (2)
C13A—C14A—H14A	119.3	N1C—C4C—H4CA	109.5
C14A—C15A—C16A	118.7 (3)	C3C—C4C—H4CA	109.5
C14A—C15A—H15A	120.6	N1C—C4C—H4CB	109.5
C16A—C15A—H15A	120.6	C3C—C4C—H4CB	109.5
C17A—C16A—C15A	120.9 (2)	H4CA—C4C—H4CB	108.1
C17A—C16A—I1A	119.64 (19)	C6C—C5C—C10C	117.7 (2)
C15A—C16A—I1A	119.5 (2)	C6C—C5C—N2C	119.2 (2)
C16A—C17A—C18A	119.7 (2)	C10C—C5C—N2C	123.0 (2)
C16A—C17A—H17A	120.2	C7C—C6C—C5C	121.4 (2)
C18A—C17A—H17A	120.2	C7C—C6C—H6C	119.3
C17A—C18A—C13A	120.5 (2)	C5C—C6C—H6C	119.3
C17A—C18A—H18A	119.7	C6C—C7C—C8C	120.5 (2)
C13A—C18A—H18A	119.7	C6C—C7C—H7C	119.8
C1B—N1B—C4B	109.9 (2)	C8C—C7C—H7C	119.8
C1B—N1B—H1BA	111.2 (18)	O1C—C8C—C9C	116.5 (2)
C4B—N1B—H1BA	110.7 (18)	O1C—C8C—C7C	125.2 (2)
C1B—N1B—H1BB	113.3 (16)	C9C—C8C—C7C	118.3 (2)
C4B—N1B—H1BB	105.8 (17)	C10C—C9C—C8C	121.3 (3)
H1BA—N1B—H1BB	106 (2)	C10C—C9C—H9C	119.3
C5B—N2B—C2B	114.91 (19)	C8C—C9C—H9C	119.3
C5B—N2B—C3B	115.9 (2)	C9C—C10C—C5C	120.8 (2)
C2B—N2B—C3B	110.5 (2)	C9C—C10C—H10C	119.6
N1B—C1B—C2B	109.5 (2)	C5C—C10C—H10C	119.6
N1B—C1B—H1BC	109.8	C8C—O1C—C11C	117.0 (2)
C2B—C1B—H1BC	109.8	O1C—C11C—H11G	109.5
N1B—C1B—H1BD	109.8	O1C—C11C—H11H	109.5
C2B—C1B—H1BD	109.8	H11G—C11C—H11H	109.5
H1BC—C1B—H1BD	108.2	O1C—C11C—H11I	109.5
N2B—C2B—C1B	111.0 (2)	H11G—C11C—H11I	109.5
N2B—C2B—H2BA	109.4	H11H—C11C—H11I	109.5
C1B—C2B—H2BA	109.4	O3C—C12C—O2C	123.7 (2)
N2B—C2B—H2BB	109.4	O3C—C12C—C13C	118.7 (2)
C1B—C2B—H2BB	109.4	O2C—C12C—C13C	117.6 (2)
H2BA—C2B—H2BB	108.0	C14C—C13C—C18C	118.5 (2)
N2B—C3B—C4B	111.4 (2)	C14C—C13C—C12C	120.4 (2)
N2B—C3B—H3B1	109.3	C18C—C13C—C12C	121.1 (2)
C4B—C3B—H3B1	109.3	C15C—C14C—C13C	121.4 (2)
N2B—C3B—H3B2	109.3	C15C—C14C—H14C	119.3
C4B—C3B—H3B2	109.3	C13C—C14C—H14C	119.3
H3B1—C3B—H3B2	108.0	C14C—C15C—C16C	118.5 (3)
N1B—C4B—C3B	110.4 (2)	C14C—C15C—H15C	120.8
N1B—C4B—H4BA	109.6	C16C—C15C—H15C	120.8
C3B—C4B—H4BA	109.6	C17C—C16C—C15C	121.5 (3)
N1B—C4B—H4BB	109.6	C17C—C16C—I1C	119.61 (19)
C3B—C4B—H4BB	109.6	C15C—C16C—I1C	118.9 (2)
H4BA—C4B—H4BB	108.1	C16C—C17C—C18C	119.3 (2)
C10B—C5B—C6B	116.8 (2)	C16C—C17C—H17C	120.3
C10B—C5B—N2B	123.1 (2)	C18C—C17C—H17C	120.3
C6B—C5B—N2B	120.0 (2)	C17C—C18C—C13C	120.7 (3)
C7B—C6B—C5B	122.2 (2)	C17C—C18C—H18C	119.6
C7B—C6B—H6B	118.9	C13C—C18C—H18C	119.6
			
C4A—N1A—C1A—C2A	−59.7 (3)	C8B—C9B—C10B—C5B	0.9 (4)
C5A—N2A—C2A—C1A	173.1 (2)	C6B—C5B—C10B—C9B	−1.7 (4)
C3A—N2A—C2A—C1A	−55.1 (3)	N2B—C5B—C10B—C9B	174.0 (2)
N1A—C1A—C2A—N2A	58.0 (3)	C7B—C8B—O1B—C11B	−1.3 (4)
C5A—N2A—C3A—C4A	−175.2 (2)	C9B—C8B—O1B—C11B	179.4 (2)
C2A—N2A—C3A—C4A	53.5 (3)	O3B—C12B—C13B—C14B	6.7 (4)
C1A—N1A—C4A—C3A	58.9 (3)	O2B—C12B—C13B—C14B	−172.3 (2)
N2A—C3A—C4A—N1A	−55.7 (3)	O3B—C12B—C13B—C18B	−173.8 (2)
C3A—N2A—C5A—C10A	1.4 (3)	O2B—C12B—C13B—C18B	7.1 (3)
C2A—N2A—C5A—C10A	131.8 (3)	C18B—C13B—C14B—C15B	−1.6 (4)
C3A—N2A—C5A—C6A	179.3 (2)	C12B—C13B—C14B—C15B	177.9 (2)
C2A—N2A—C5A—C6A	−50.3 (3)	C13B—C14B—C15B—C16B	0.9 (4)
C10A—C5A—C6A—C7A	−0.3 (4)	C14B—C15B—C16B—C17B	0.4 (4)
N2A—C5A—C6A—C7A	−178.3 (2)	C14B—C15B—C16B—I1B	−178.92 (18)
C5A—C6A—C7A—C8A	0.6 (4)	C15B—C16B—C17B—C18B	−1.1 (4)
C6A—C7A—C8A—O1A	−179.6 (2)	I1B—C16B—C17B—C18B	178.25 (19)
C6A—C7A—C8A—C9A	−0.5 (4)	C16B—C17B—C18B—C13B	0.4 (4)
O1A—C8A—C9A—C10A	179.3 (2)	C14B—C13B—C18B—C17B	0.9 (4)
C7A—C8A—C9A—C10A	0.1 (4)	C12B—C13B—C18B—C17B	−178.6 (2)
C8A—C9A—C10A—C5A	0.2 (4)	C4C—N1C—C1C—C2C	−55.2 (3)
C6A—C5A—C10A—C9A	−0.1 (4)	C5C—N2C—C2C—C1C	166.3 (2)
N2A—C5A—C10A—C9A	177.8 (2)	C3C—N2C—C2C—C1C	−59.0 (3)
C7A—C8A—O1A—C11A	−5.2 (4)	N1C—C1C—C2C—N2C	57.1 (3)
C9A—C8A—O1A—C11A	175.6 (2)	C5C—N2C—C3C—C4C	−167.4 (2)
O3A—C12A—C13A—C14A	3.8 (4)	C2C—N2C—C3C—C4C	58.4 (3)
O2A—C12A—C13A—C14A	−176.1 (2)	C1C—N1C—C4C—C3C	55.1 (3)
O3A—C12A—C13A—C18A	−176.6 (2)	N2C—C3C—C4C—N1C	−56.4 (3)
O2A—C12A—C13A—C18A	3.5 (4)	C2C—N2C—C5C—C6C	−49.1 (3)
C18A—C13A—C14A—C15A	−0.5 (4)	C3C—N2C—C5C—C6C	178.6 (2)
C12A—C13A—C14A—C15A	179.1 (2)	C2C—N2C—C5C—C10C	135.7 (2)
C13A—C14A—C15A—C16A	0.6 (4)	C3C—N2C—C5C—C10C	3.5 (3)
C14A—C15A—C16A—C17A	−0.4 (4)	C10C—C5C—C6C—C7C	0.6 (4)
C14A—C15A—C16A—I1A	−179.42 (19)	N2C—C5C—C6C—C7C	−174.8 (2)
C15A—C16A—C17A—C18A	0.1 (4)	C5C—C6C—C7C—C8C	1.3 (4)
I1A—C16A—C17A—C18A	179.17 (19)	C6C—C7C—C8C—O1C	177.9 (2)
C16A—C17A—C18A—C13A	−0.1 (4)	C6C—C7C—C8C—C9C	−1.9 (4)
C14A—C13A—C18A—C17A	0.3 (4)	O1C—C8C—C9C—C10C	−179.2 (2)
C12A—C13A—C18A—C17A	−179.3 (2)	C7C—C8C—C9C—C10C	0.6 (4)
C4B—N1B—C1B—C2B	−58.0 (3)	C8C—C9C—C10C—C5C	1.3 (4)
C5B—N2B—C2B—C1B	168.0 (2)	C6C—C5C—C10C—C9C	−1.9 (4)
C3B—N2B—C2B—C1B	−58.5 (3)	N2C—C5C—C10C—C9C	173.3 (2)
N1B—C1B—C2B—N2B	59.4 (3)	C9C—C8C—O1C—C11C	172.3 (2)
C5B—N2B—C3B—C4B	−170.5 (2)	C7C—C8C—O1C—C11C	−7.6 (4)
C2B—N2B—C3B—C4B	56.6 (3)	O3C—C12C—C13C—C14C	22.7 (4)
C1B—N1B—C4B—C3B	56.4 (3)	O2C—C12C—C13C—C14C	−157.3 (2)
N2B—C3B—C4B—N1B	−55.9 (3)	O3C—C12C—C13C—C18C	−156.5 (2)
C2B—N2B—C5B—C10B	137.5 (2)	O2C—C12C—C13C—C18C	23.6 (3)
C3B—N2B—C5B—C10B	6.6 (3)	C18C—C13C—C14C—C15C	0.2 (4)
C2B—N2B—C5B—C6B	−46.9 (3)	C12C—C13C—C14C—C15C	−179.0 (2)
C3B—N2B—C5B—C6B	−177.8 (2)	C13C—C14C—C15C—C16C	1.8 (4)
C10B—C5B—C6B—C7B	1.1 (4)	C14C—C15C—C16C—C17C	−2.1 (4)
N2B—C5B—C6B—C7B	−174.8 (2)	C14C—C15C—C16C—I1C	176.51 (19)
C5B—C6B—C7B—C8B	0.4 (4)	C15C—C16C—C17C—C18C	0.5 (4)
C6B—C7B—C8B—O1B	179.5 (2)	I1C—C16C—C17C—C18C	−178.12 (19)
C6B—C7B—C8B—C9B	−1.3 (4)	C16C—C17C—C18C—C13C	1.5 (4)
O1B—C8B—C9B—C10B	180.0 (2)	C14C—C13C—C18C—C17C	−1.8 (4)
C7B—C8B—C9B—C10B	0.6 (4)	C12C—C13C—C18C—C17C	177.3 (2)

### 4-(4-Methoxyphenyl)piperazin-1-ium 4-iodobenzoate monohydrate (III) Hydrogen-bond geometry (Å, º)

**Table:**

*D*—H···*A*	*D*—H	H···*A*	*D*···*A*	*D*—H···*A*
O1*W*—H1*W*1···O3*C*^i^	0.77 (2)	1.93 (2)	2.696 (3)	173 (3)
O1*W*—H2*W*1···O2*A*	0.77 (2)	1.96 (2)	2.700 (3)	162 (3)
O2*W*—H1*W*2···O3*A*	0.77 (1)	1.86 (2)	2.626 (2)	170 (3)
O2*W*—H2*W*2···O2*B*	0.77 (2)	1.97 (2)	2.733 (3)	168 (3)
O3*W*—H1*W*3···O3*B*	0.77 (2)	1.87 (2)	2.636 (2)	172 (3)
O3*W*—H2*W*3···O2*C*	0.78 (1)	1.94 (2)	2.706 (3)	172 (3)
N1*A*—H1*AB*···O3*W*^ii^	0.94 (3)	1.82 (3)	2.754 (3)	172 (3)
N1*A*—H1*AA*···O2*A*	0.90 (3)	1.89 (3)	2.776 (3)	168 (3)
C1*A*—H1*AC*···O2*W*	0.99	2.57	3.362 (3)	136
C2*A*—H2*AB*···O3*B*^ii^	0.99	2.51	3.498 (3)	175
C4*A*—H4*AA*···O2*C*^iii^	0.99	2.47	3.441 (3)	166
C7*A*—H7*A*···I1*B*^iii^	0.95	3.32	4.212 (2)	156
C11*A*—H11*A*···O1*B*^iv^	0.98	2.51	3.225 (4)	130
N1*B*—H1*BA*···O2*B*	0.91 (3)	1.87 (3)	2.780 (3)	174 (3)
N1*B*—H1*BB*···O2*W*^ii^	0.92 (3)	1.85 (3)	2.768 (3)	176 (2)
C1*B*—H1*BC*···O3*W*	0.99	2.44	3.229 (3)	136
C2*B*—H2*BB*···O3*A*^ii^	0.99	2.63	3.619 (3)	174
C4*B*—H4*BA*···O2*B*^iii^	0.99	2.51	3.491 (3)	170
C4*B*—H4*BB*···O2*W*	0.99	2.52	3.260 (3)	131
C9*B*—H9*B*···I1*B*^ii^	0.95	3.25	4.020 (3)	139
N1*C*—H1*CA*···O2*C*	0.96 (3)	1.78 (3)	2.732 (3)	172 (3)
N1*C*—H1*CA*···O3*C*	0.96 (3)	2.64 (3)	3.297 (3)	126 (2)
N1*C*—H1*CB*···O1*W*^ii^	0.83 (3)	1.96 (3)	2.781 (3)	172 (3)
C1*C*—H1*CC*···O1*W*^v^	0.99	2.39	3.299 (3)	152
C4*C*—H4*CA*···O2*A*^iii^	0.99	2.45	3.438 (3)	174
C9*C*—H9*C*···I1*A*^ii^	0.95	3.29	4.013 (3)	135

Symmetry codes: (i) *x*, −*y*+1/2, *z*+1/2; (ii) −*x*+1, −*y*+1, −*z*+1; (iii) −*x*+1, −*y*, −*z*+1; (iv) −*x*+2, −*y*+1, −*z*+1; (v) *x*, −*y*+1/2, *z*−1/2.

### 4-(4-Methoxyphenyl)piperazin-1-ium 4-methylbenzoate monohydrate (IV) Crystal data

**Table:**

C_11_H_17_N_2_O^+^·C_8_H_7_O_2_^−^·H_2_O	*Z* = 2
*M**_r_* = 346.42	*F*(000) = 372
Triclinic, *P*1	*D*_x_ = 1.305 Mg m^−^^3^
*a* = 6.1481 (13) Å	Mo *K*α radiation, λ = 0.71073 Å
*b* = 7.3467 (12) Å	Cell parameters from 9904 reflections
*c* = 19.980 (4) Å	θ = 2.8–27.5°
α = 80.190 (6)°	µ = 0.09 mm^−^^1^
β = 86.089 (5)°	*T* = 90 K
γ = 82.843 (6)°	Plate, colourless
*V* = 881.3 (3) Å^3^	0.29 × 0.21 × 0.02 mm

### 4-(4-Methoxyphenyl)piperazin-1-ium 4-methylbenzoate monohydrate (IV) Data collection

**Table:**

Bruker D8 Venture dual source diffractometer	4059 independent reflections
Radiation source: microsource	3137 reflections with *I* > 2σ(*I*)
Detector resolution: 7.41 pixels mm^-1^	*R*_int_ = 0.041
φ and ω scans	θ_max_ = 27.6°, θ_min_ = 2.1°
Absorption correction: multi-scan (*SADABS*; Krause *et al*., 2015)	*h* = −7→7
*T*_min_ = 0.877, *T*_max_ = 0.959	*k* = −9→9
23917 measured reflections	*l* = −25→25

### 4-(4-Methoxyphenyl)piperazin-1-ium 4-methylbenzoate monohydrate (IV) Refinement

**Table:**

Refinement on *F*^2^	Secondary atom site location: difference Fourier map
Least-squares matrix: full	Hydrogen site location: mixed
*R*[*F*^2^ > 2σ(*F*^2^)] = 0.038	H atoms treated by a mixture of independent and constrained refinement
*wR*(*F*^2^) = 0.080	*w* = 1/[σ^2^(*F*_o_^2^) + (0.0124*P*)^2^ + 0.3732*P*] where *P* = (*F*_o_^2^ + 2*F*_c_^2^)/3
*S* = 1.07	(Δ/σ)_max_ = 0.001
4059 reflections	Δρ_max_ = 0.19 e Å^−^^3^
245 parameters	Δρ_min_ = −0.18 e Å^−^^3^
0 restraints	Extinction correction: SHELXL-2019/2 (Sheldrick 2015b), Fc^*^=kFc[1+0.001xFc^2^λ^3^/sin(2θ)]^-1/4^
Primary atom site location: structure-invariant direct methods	Extinction coefficient: 0.0038 (8)

### 4-(4-Methoxyphenyl)piperazin-1-ium 4-methylbenzoate monohydrate (IV) Special details

**Table:**

Experimental. The crystal was mounted using polyisobutene oil on the tip of a fine glass fibre, which was fastened in a copper mounting pin with electrical solder. It was placed directly into the cold gas stream of a liquid-nitrogen based cryostat (Hope, 1994; Parkin & Hope, 1998). Diffraction data were collected with the crystal at 90K, which is standard practice in this laboratory for the majority of flash-cooled crystals.
Geometry. All esds (except the esd in the dihedral angle between two l.s. planes) are estimated using the full covariance matrix. The cell esds are taken into account individually in the estimation of esds in distances, angles and torsion angles; correlations between esds in cell parameters are only used when they are defined by crystal symmetry. An approximate (isotropic) treatment of cell esds is used for estimating esds involving l.s. planes.
Refinement. Refinement progress was checked using *Platon* (Spek, 2020) and by an *R*-tensor (Parkin, 2000). The final model was further checked with the IUCr utility *checkCIF*.

### 4-(4-Methoxyphenyl)piperazin-1-ium 4-methylbenzoate monohydrate (IV) Fractional atomic coordinates and isotropic or equivalent isotropic displacement parameters (Å^2^)

**Table:**

	*x*	*y*	*z*	*U*_iso_*/*U*_eq_	
N1	0.23887 (18)	0.74787 (16)	0.54355 (5)	0.0192 (2)	
H1A	0.203 (3)	0.727 (2)	0.4961 (9)	0.042 (5)*	
H1B	0.271 (3)	0.871 (2)	0.5385 (8)	0.033 (4)*	
N2	0.28444 (17)	0.65390 (15)	0.68884 (5)	0.0179 (2)	
C1	0.4273 (2)	0.61887 (18)	0.57271 (6)	0.0202 (3)	
H1C	0.558241	0.630796	0.541287	0.024*	
H1D	0.392248	0.489067	0.578218	0.024*	
C2	0.4760 (2)	0.66315 (19)	0.64091 (6)	0.0196 (3)	
H2A	0.598899	0.573865	0.660243	0.024*	
H2B	0.522549	0.789461	0.634542	0.024*	
C3	0.0887 (2)	0.76711 (19)	0.66009 (6)	0.0211 (3)	
H3A	0.108509	0.900258	0.656176	0.025*	
H3B	−0.039758	0.743193	0.691596	0.025*	
C4	0.0427 (2)	0.7273 (2)	0.59089 (6)	0.0220 (3)	
H4A	0.002660	0.599190	0.595399	0.026*	
H4B	−0.082879	0.814471	0.572187	0.026*	
C5	0.3246 (2)	0.68052 (18)	0.75566 (6)	0.0183 (3)	
C6	0.5285 (2)	0.62481 (19)	0.78350 (6)	0.0227 (3)	
H6	0.644111	0.569862	0.756838	0.027*	
C7	0.5680 (2)	0.6472 (2)	0.84923 (7)	0.0254 (3)	
H7	0.709269	0.608607	0.866686	0.030*	
C8	0.4021 (2)	0.7252 (2)	0.88893 (6)	0.0240 (3)	
C9	0.1971 (2)	0.7774 (2)	0.86282 (7)	0.0274 (3)	
H9	0.080563	0.827557	0.890402	0.033*	
C10	0.1588 (2)	0.7577 (2)	0.79726 (7)	0.0252 (3)	
H10	0.017294	0.797357	0.780137	0.030*	
O1	0.42146 (17)	0.75852 (16)	0.95411 (5)	0.0337 (3)	
C11	0.6322 (3)	0.7136 (3)	0.98204 (7)	0.0392 (4)	
H11A	0.623438	0.740327	1.028619	0.059*	
H11B	0.737047	0.788298	0.954254	0.059*	
H11C	0.681120	0.581268	0.982463	0.059*	
O2	0.12234 (15)	0.71150 (13)	0.41700 (4)	0.0241 (2)	
O3	0.39970 (17)	0.87663 (14)	0.37969 (5)	0.0321 (3)	
C12	0.2413 (2)	0.79566 (18)	0.36975 (7)	0.0206 (3)	
C13	0.1882 (2)	0.79539 (18)	0.29744 (6)	0.0190 (3)	
C14	0.3302 (2)	0.86250 (19)	0.24411 (7)	0.0229 (3)	
H14	0.457991	0.912589	0.253423	0.027*	
C15	0.2855 (2)	0.8564 (2)	0.17733 (7)	0.0259 (3)	
H15	0.384517	0.901179	0.141471	0.031*	
C16	0.0986 (2)	0.78599 (19)	0.16202 (7)	0.0241 (3)	
C17	−0.0448 (2)	0.72276 (19)	0.21558 (7)	0.0226 (3)	
H17	−0.174944	0.676288	0.206177	0.027*	
C18	−0.0004 (2)	0.72670 (18)	0.28226 (6)	0.0201 (3)	
H18	−0.099671	0.682046	0.318066	0.024*	
C19	0.0474 (3)	0.7789 (2)	0.08986 (7)	0.0353 (4)	
H19A	−0.003498	0.658960	0.087676	0.053*	
H19B	0.179989	0.793648	0.060251	0.053*	
H19C	−0.067539	0.879581	0.074736	0.053*	
O1W	0.72107 (18)	0.87492 (14)	0.46115 (5)	0.0241 (2)	
H1W	0.600 (3)	0.874 (3)	0.4328 (10)	0.062 (6)*	
H2W	0.850 (3)	0.813 (3)	0.4429 (10)	0.060 (6)*	

### 4-(4-Methoxyphenyl)piperazin-1-ium 4-methylbenzoate monohydrate (IV) Atomic displacement parameters (Å^2^)

**Table:**

	*U*^11^	*U*^22^	*U*^33^	*U*^12^	*U*^13^	*U*^23^
N1	0.0202 (6)	0.0197 (6)	0.0178 (6)	−0.0037 (5)	−0.0013 (4)	−0.0026 (4)
N2	0.0149 (5)	0.0234 (6)	0.0157 (5)	−0.0021 (4)	−0.0007 (4)	−0.0038 (4)
C1	0.0192 (7)	0.0225 (7)	0.0187 (6)	−0.0006 (5)	−0.0003 (5)	−0.0045 (5)
C2	0.0162 (6)	0.0244 (7)	0.0184 (6)	−0.0033 (5)	0.0006 (5)	−0.0034 (5)
C3	0.0156 (6)	0.0276 (7)	0.0199 (7)	−0.0012 (6)	−0.0008 (5)	−0.0037 (5)
C4	0.0175 (7)	0.0277 (7)	0.0208 (7)	−0.0037 (6)	−0.0009 (5)	−0.0026 (5)
C5	0.0198 (7)	0.0187 (7)	0.0166 (6)	−0.0047 (5)	0.0001 (5)	−0.0020 (5)
C6	0.0188 (7)	0.0298 (8)	0.0197 (7)	−0.0016 (6)	0.0009 (5)	−0.0059 (5)
C7	0.0191 (7)	0.0358 (8)	0.0214 (7)	−0.0026 (6)	−0.0041 (5)	−0.0043 (6)
C8	0.0269 (8)	0.0303 (8)	0.0160 (6)	−0.0056 (6)	−0.0009 (5)	−0.0059 (5)
C9	0.0250 (7)	0.0362 (9)	0.0210 (7)	0.0008 (6)	0.0024 (6)	−0.0092 (6)
C10	0.0197 (7)	0.0334 (8)	0.0224 (7)	−0.0002 (6)	−0.0020 (5)	−0.0064 (6)
O1	0.0288 (6)	0.0545 (7)	0.0199 (5)	−0.0010 (5)	−0.0044 (4)	−0.0138 (5)
C11	0.0319 (9)	0.0664 (12)	0.0227 (8)	−0.0079 (8)	−0.0061 (6)	−0.0135 (7)
O2	0.0261 (5)	0.0270 (5)	0.0194 (5)	−0.0023 (4)	−0.0018 (4)	−0.0044 (4)
O3	0.0324 (6)	0.0365 (6)	0.0310 (6)	−0.0122 (5)	−0.0115 (5)	−0.0059 (4)
C12	0.0207 (7)	0.0171 (7)	0.0243 (7)	0.0025 (5)	−0.0051 (5)	−0.0061 (5)
C13	0.0187 (6)	0.0185 (7)	0.0198 (6)	0.0008 (5)	−0.0025 (5)	−0.0045 (5)
C14	0.0176 (7)	0.0236 (7)	0.0283 (7)	−0.0037 (6)	−0.0015 (5)	−0.0059 (6)
C15	0.0241 (7)	0.0307 (8)	0.0218 (7)	−0.0031 (6)	0.0029 (5)	−0.0032 (6)
C16	0.0266 (7)	0.0261 (7)	0.0195 (7)	0.0009 (6)	−0.0033 (5)	−0.0055 (5)
C17	0.0216 (7)	0.0234 (7)	0.0244 (7)	−0.0034 (6)	−0.0048 (5)	−0.0061 (5)
C18	0.0194 (7)	0.0195 (7)	0.0210 (7)	−0.0020 (5)	−0.0006 (5)	−0.0027 (5)
C19	0.0397 (9)	0.045 (1)	0.0223 (7)	−0.0043 (8)	−0.0038 (7)	−0.0080 (7)
O1W	0.0229 (5)	0.0260 (5)	0.0245 (5)	−0.0045 (4)	−0.0043 (4)	−0.0045 (4)

### 4-(4-Methoxyphenyl)piperazin-1-ium 4-methylbenzoate monohydrate (IV) Geometric parameters (Å, º)

**Table:**

N1—C1	1.4864 (17)	C9—H9	0.9500
N1—C4	1.4879 (17)	C10—H10	0.9500
N1—H1A	1.028 (17)	O1—C11	1.4242 (18)
N1—H1B	0.939 (16)	C11—H11A	0.9800
N2—C5	1.4239 (16)	C11—H11B	0.9800
N2—C3	1.4669 (16)	C11—H11C	0.9800
N2—C2	1.4674 (16)	O2—C12	1.2759 (16)
C1—C2	1.5111 (17)	O3—C12	1.2461 (16)
C1—H1C	0.9900	C12—C13	1.5032 (18)
C1—H1D	0.9900	C13—C18	1.3917 (18)
C2—H2A	0.9900	C13—C14	1.3935 (18)
C2—H2B	0.9900	C14—C15	1.3897 (19)
C3—C4	1.5131 (18)	C14—H14	0.9500
C3—H3A	0.9900	C15—C16	1.3894 (19)
C3—H3B	0.9900	C15—H15	0.9500
C4—H4A	0.9900	C16—C17	1.3937 (19)
C4—H4B	0.9900	C16—C19	1.5074 (19)
C5—C6	1.3918 (18)	C17—C18	1.3840 (18)
C5—C10	1.3974 (18)	C17—H17	0.9500
C6—C7	1.3920 (18)	C18—H18	0.9500
C6—H6	0.9500	C19—H19A	0.9800
C7—C8	1.3777 (19)	C19—H19B	0.9800
C7—H7	0.9500	C19—H19C	0.9800
C8—O1	1.3809 (16)	O1W—H1W	0.97 (2)
C8—C9	1.381 (2)	O1W—H2W	0.94 (2)
C9—C10	1.3809 (19)		
			
C1—N1—C4	109.09 (10)	C7—C8—C9	118.96 (12)
C1—N1—H1A	113.3 (9)	O1—C8—C9	115.52 (12)
C4—N1—H1A	109.7 (9)	C10—C9—C8	121.06 (13)
C1—N1—H1B	109.9 (10)	C10—C9—H9	119.5
C4—N1—H1B	108.5 (10)	C8—C9—H9	119.5
H1A—N1—H1B	106.2 (13)	C9—C10—C5	121.16 (13)
C5—N2—C3	114.15 (10)	C9—C10—H10	119.4
C5—N2—C2	114.57 (10)	C5—C10—H10	119.4
C3—N2—C2	112.07 (10)	C8—O1—C11	117.39 (11)
N1—C1—C2	110.21 (11)	O1—C11—H11A	109.5
N1—C1—H1C	109.6	O1—C11—H11B	109.5
C2—C1—H1C	109.6	H11A—C11—H11B	109.5
N1—C1—H1D	109.6	O1—C11—H11C	109.5
C2—C1—H1D	109.6	H11A—C11—H11C	109.5
H1C—C1—H1D	108.1	H11B—C11—H11C	109.5
N2—C2—C1	111.9 (1)	O3—C12—O2	124.23 (12)
N2—C2—H2A	109.2	O3—C12—C13	117.92 (12)
C1—C2—H2A	109.2	O2—C12—C13	117.85 (11)
N2—C2—H2B	109.2	C18—C13—C14	118.71 (12)
C1—C2—H2B	109.2	C18—C13—C12	121.19 (11)
H2A—C2—H2B	107.9	C14—C13—C12	120.10 (12)
N2—C3—C4	112.90 (11)	C15—C14—C13	120.20 (12)
N2—C3—H3A	109.0	C15—C14—H14	119.9
C4—C3—H3A	109.0	C13—C14—H14	119.9
N2—C3—H3B	109.0	C16—C15—C14	121.25 (13)
C4—C3—H3B	109.0	C16—C15—H15	119.4
H3A—C3—H3B	107.8	C14—C15—H15	119.4
N1—C4—C3	110.50 (11)	C15—C16—C17	118.16 (12)
N1—C4—H4A	109.5	C15—C16—C19	121.89 (13)
C3—C4—H4A	109.5	C17—C16—C19	119.95 (13)
N1—C4—H4B	109.5	C18—C17—C16	120.95 (12)
C3—C4—H4B	109.5	C18—C17—H17	119.5
H4A—C4—H4B	108.1	C16—C17—H17	119.5
C6—C5—C10	116.85 (12)	C17—C18—C13	120.71 (12)
C6—C5—N2	121.63 (12)	C17—C18—H18	119.6
C10—C5—N2	121.48 (12)	C13—C18—H18	119.6
C5—C6—C7	122.01 (13)	C16—C19—H19A	109.5
C5—C6—H6	119.0	C16—C19—H19B	109.5
C7—C6—H6	119.0	H19A—C19—H19B	109.5
C8—C7—C6	119.92 (13)	C16—C19—H19C	109.5
C8—C7—H7	120.0	H19A—C19—H19C	109.5
C6—C7—H7	120.0	H19B—C19—H19C	109.5
C7—C8—O1	125.52 (12)	H1W—O1W—H2W	109.5 (16)
			
C4—N1—C1—C2	−59.83 (13)	C8—C9—C10—C5	1.5 (2)
C5—N2—C2—C1	175.50 (11)	C6—C5—C10—C9	0.1 (2)
C3—N2—C2—C1	−52.38 (14)	N2—C5—C10—C9	177.80 (13)
N1—C1—C2—N2	57.29 (14)	C7—C8—O1—C11	2.0 (2)
C5—N2—C3—C4	−176.52 (11)	C9—C8—O1—C11	−177.61 (14)
C2—N2—C3—C4	51.15 (14)	O3—C12—C13—C18	−172.58 (12)
C1—N1—C4—C3	58.20 (14)	O2—C12—C13—C18	8.03 (18)
N2—C3—C4—N1	−54.40 (15)	O3—C12—C13—C14	8.23 (19)
C3—N2—C5—C6	−162.71 (12)	O2—C12—C13—C14	−171.15 (12)
C2—N2—C5—C6	−31.58 (17)	C18—C13—C14—C15	−1.47 (19)
C3—N2—C5—C10	19.66 (17)	C12—C13—C14—C15	177.74 (12)
C2—N2—C5—C10	150.78 (12)	C13—C14—C15—C16	0.8 (2)
C10—C5—C6—C7	−1.0 (2)	C14—C15—C16—C17	0.6 (2)
N2—C5—C6—C7	−178.78 (12)	C14—C15—C16—C19	179.81 (14)
C5—C6—C7—C8	0.5 (2)	C15—C16—C17—C18	−1.2 (2)
C6—C7—C8—O1	−178.52 (13)	C19—C16—C17—C18	179.54 (13)
C6—C7—C8—C9	1.1 (2)	C16—C17—C18—C13	0.5 (2)
C7—C8—C9—C10	−2.0 (2)	C14—C13—C18—C17	0.85 (19)
O1—C8—C9—C10	177.58 (13)	C12—C13—C18—C17	−178.35 (12)

### 4-(4-Methoxyphenyl)piperazin-1-ium 4-methylbenzoate monohydrate (IV) Hydrogen-bond geometry (Å, º)

**Table:**

*D*—H···*A*	*D*—H	H···*A*	*D*···*A*	*D*—H···*A*
N1—H1*A*···O2	1.028 (17)	1.714 (17)	2.7391 (15)	174.1 (15)
N1—H1*B*···O1*W*^i^	0.939 (16)	1.873 (17)	2.7977 (16)	167.8 (14)
C1—H1*C*···O1*W*	0.99	2.46	3.2617 (17)	138
C2—H2*B*···O3^i^	0.99	2.52	3.5088 (17)	174
C4—H4*A*···O2^ii^	0.99	2.55	3.5294 (17)	169
C4—H4*B*···O1*W*^iii^	0.99	2.54	3.3184 (17)	135
O1*W*—H1*W*···O3	0.97 (2)	1.67 (2)	2.6418 (14)	176.5 (18)
O1*W*—H2*W*···O2^iv^	0.94 (2)	1.83 (2)	2.7626 (15)	170.5 (17)

Symmetry codes: (i) −*x*+1, −*y*+2, −*z*+1; (ii) −*x*, −*y*+1, −*z*+1; (iii) *x*−1, *y*, *z*; (iv) *x*+1, *y*, *z*.
